# Human RAD18 Interacts with Ubiquitylated Chromatin Components and Facilitates RAD9 Recruitment to DNA Double Strand Breaks

**DOI:** 10.1371/journal.pone.0023155

**Published:** 2011-08-17

**Authors:** Akiko Inagaki, Esther Sleddens-Linkels, Wiggert A. van Cappellen, Richard G. Hibbert, Titia K. Sixma, Jan H. J. Hoeijmakers, J. Anton Grootegoed, Willy M. Baarends

**Affiliations:** 1 Department of Reproduction and Development, Erasmus Medical Center - University Medical Center, Rotterdam, The Netherlands; 2 Department of Cell Biology and Genetics, Erasmus Medical Center - University Medical Center, Rotterdam, The Netherlands; 3 Division of Biochemistry and Center for Biomedical Genetics, Netherlands Cancer Institute, Amsterdam, The Netherlands; University of Minnesota, United States of America

## Abstract

RAD18 is an ubiquitin ligase involved in replicative damage bypass and DNA double-strand break (DSB) repair processes. We found that RPA is required for the dynamic pattern of RAD18 localization during the cell cycle, and for accumulation of RAD18 at sites of γ-irradiation-induced DNA damage. In addition, RAD18 colocalizes with chromatin-associated conjugated ubiquitin and ubiquitylated H2A throughout the cell cycle and following irradiation. This localization pattern depends on the presence of an intact, ubiquitin-binding Zinc finger domain. Using a biochemical approach, we show that RAD18 directly binds to ubiquitylated H2A and several other unknown ubiquitylated chromatin components. This interaction also depends on the RAD18 Zinc finger, and increases upon the induction of DSBs by γ-irradiation. Intriguingly, RAD18 does not always colocalize with regions that show enhanced H2A ubiquitylation. In human female primary fibroblasts, where one of the two X chromosomes is inactivated to equalize X-chromosomal gene expression between male (XY) and female (XX) cells, this inactive X is enriched for ubiquitylated H2A, but only rarely accumulates RAD18. This indicates that the binding of RAD18 to ubiquitylated H2A is context-dependent. Regarding the functional relevance of RAD18 localization at DSBs, we found that RAD18 is required for recruitment of RAD9, one of the components of the 9-1-1 checkpoint complex, to these sites. Recruitment of RAD9 requires the functions of the RING and Zinc finger domains of RAD18. Together, our data indicate that association of RAD18 with DSBs through ubiquitylated H2A and other ubiquitylated chromatin components allows recruitment of RAD9, which may function directly in DSB repair, independent of downstream activation of the checkpoint kinases CHK1 and CHK2.

## Introduction

Mammalian cells require the E3 ubiquitin ligase RAD18 for survival after the induction of various types of DNA damage. *RAD18* knockout cells are sensitive to UVC light exposure [1], [2], [3], camptothecin [1], [4], and ionizing radiation (IR) [1], [4], [5], that induce distortions of DNA geometry, single strand breaks (SSBs), and double strand breaks (DSBs), respectively. RAD18 complexes with the two mammalian orthologs of the yeast E2 ubiquitin-conjugating enzyme Rad6; HR6A (UBE2A) and HR6B (UBE2B) [6]. Rad6 is most well known for its role in replicative damage bypass (RDB) that allows progression of DNA replication in the presence of DNA damage (reviewed in [7]). The first step in the RDB pathway involves mono-ubiquitylation of PCNA by the RAD18-HR6A/B complex [8]. PCNA forms a homotrimer that encircles double-stranded DNA, and operates as a sliding clamp to keep the DNA polymerase machinery firmly on the DNA during DNA replication (reviewed in [9]). Mono-ubiquitylation of PCNA by the RAD18-HR6A/B complex recruits specific translesion synthesis polymerases that can incorporate nucleotides in the strand opposite the site of the DNA lesions [10]. RAD18 contains a RING finger that has been shown to be required for ubiquitylation of PCNA [1], [10]. In addition to this domain, HR6A/B interacting domains [11], [12], [13], and a so-called SAP domain that shows binding affinity to single-stranded DNA (ssDNA) *in vitro* [14] have been identified. The SAP domain is also required for PCNA ubiquitylation [1]. Finally, it was recently described that RAD18 also contains a Zinc finger that functions as an ubiquitin binding domain [14], [15], [16], [17].

In addition to the RDB pathway, RAD18 also functions in DSB repair. DSBs may arise from exogenous factors such as ionizing radiation. In addition, DSBs can arise when the replication fork collapses during S phase. Two distinct DSB repair pathways have been identified in mammalian cells; non-homologous end-joining (NHEJ), and homologous recombination (HR). NHEJ is an error-prone form of DSB repair, in which the two ends of the broken DNA are processed for direct ligation. This mechanism is thought to be operative mainly during the G1 phase. In contrast, HR is an error-free mechanism, in which a homologous sequence of the sister chromatid is used as a template to process repair in S and G2 phases. All HR pathways are initiated by 5′-3′ degradation of one strand at both sides of the break; the so-called DNA-end resection, generating stretches of ssDNA, that are subsequently coated by the ssDNA binding protein complex RPA (reviewed in [18]). RPA is a heterotrimeric protein complex composed of RPA1, RPA2 and RPA3, and is essential for DNA replication and various DNA repair pathways [18], [19], [20], [21], [22], [23]. RPA is subsequently replaced by RAD51 on the single stranded tails, which allows efficient RAD51-mediated recombination [24]. Previous analyses in *S. cerevisiae* and human cells have shown that RAD18 interacts directly with RPA [25], [26]. Furthermore, it has been suggested that exposure of ssDNA at stalled replication forks results in accumulation of RPA, which is essential for PCNA ubiquitylation both in *S. cerevisiae* and mammalian cells [25], [27], followed by recruitment of RAD18 [26].

RAD18 accumulates at DSBs at all cell cycle phases [1], [5], [28], and this was found to be independent of PCNA [28]. In the absence of damage, RAD18 shows a dynamic localisation pattern during the cell cycle, starting in G1 with accumulation in one or two large foci, followed by a redistribution into multiple small foci in S and early G2 that largely correspond to sites of RAD51 accumulation, followed by a redistribution into the nucleoli in late G2 [28]. During S phase, RAD18 may facilitate HR independent of PCNA, by suppressing NHEJ at DSB repair sites that result from blockage of replication [29], [30]. Recently, two novel interaction partners of RAD18, RAD51C and 53BP1, were discovered [1], [5]. The interaction between RAD51C and RAD18 may promote RAD51 recruitment and thereby facilitate HR [1], whereas the interaction of RAD18 with 53BP1 most likely promotes repair of DSBs via the NHEJ pathway, since 53BP1 has been shown to inhibit resection of DNA breaks.[31]. This indicates that the functional effect of RAD18 accumulation at a DSB will depend on which other proteins are recruited and available for interaction, and this may be determined by the chromatin context.

The chromatin surrounding DSBs undergoes various modifications. One of the first modifications is phosphorylation of histone H2A variant H2AX (γH2AX) [32]. Subsequently, histone H2AX and H2A are highly ubiquitylated by the E3 ligases RNF8 [33] and RNF168 [34], [35]. Concomitantly, a number of DNA damage response factors, including RAD18, associate with chromatin enriched in γH2AX and ubiquitylated H2A/H2AX [33], [36], [37], [38], [39], [40]. The 9-1-1 complex, which consists of RAD9, RAD1 and HUS1, forms a PCNA-like heterotrimer [41], [42],[43], and functions as checkpoint protein. Cell cycle arrest is imposed by the activation of two downstream effector kinases, CHK1 and CHK2 [44]. It has been shown that the 9-1-1 complex is required to activate CHK1 to mediate UV-induced S phase arrest [45], [46], [47], [48]. The 9-1-1 complex is also loaded at DSBs after IR [46], [49], [50], and depletion of RAD9 induces hypersensitivity to IR [46], [51], [52]. In contrast to 9-1-1 complex-dependent CHK1 phosphorylation following UV, depletion of any of the 9-1-1 components does not cause impairment of either G2/M checkpoint activation [45], [47], [52] or CHK2 phosphorylation [45], [46], [48], [52] following IR, suggesting that the role of the 9-1-1 complex in DSB repair may involve a function outside the context of checkpoint activation [46], [52]. In *S. cerevisiae*, Rad18 was reported to interact with Rad17, a component of the 9-1-1 complex (an ortholog of mammalian RAD1), and to mediate its ubiquitylation [53]. However, more recent results indicate that all components of the 9-1-1 complex are ubiquitylated, but independent of RAD18, and without any effect on the DNA damage response [54].

Herein, we aim to determine how RAD18 is recruited to DSBs, and the downstream effects of RAD18 functions at DSBs. We report that RAD18 can interact directly with ubiquitylated H2A and other ubiquitylated chromatin components via its Zinc finger domain, and this binding increases upon induction of DSBs. Furthermore, RAD18 colocalizes with ubiquitylated H2A throughout the cell cycle. Finally, we reveal that RAD18 facilitates the accumulation of RAD9 at DSBs.

## Results

### Ubiquitylation of human RAD18 is independent of its own RING finger domain

**Figure 1:**
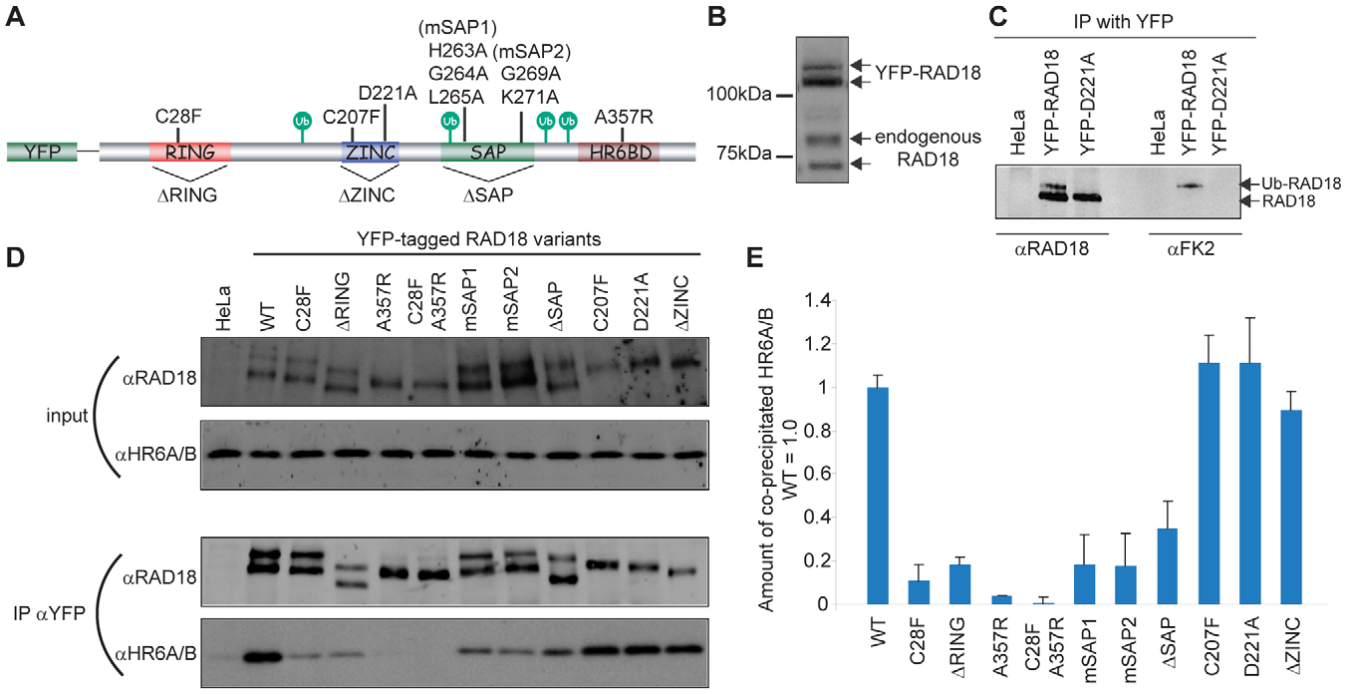
Functional analyses of human RAD18. (A) Schematic primary structure of human RAD18. All four putative ubiquitylation sites are indicated with green circles. The details of all RAD18 mutants are described in Materials and Methods. (B) Expression levels detected on immunoblots with αRAD18, for endogenous RAD18 and stably expressed YFP-RAD18 in HeLa cells. (C) HeLa cells stably expressing YFP-RAD18 or transiently expressing YFP-mutant RAD18, and wild-type HeLa cells were lysed and immunoprecipitation with YFP antibody (IP αYFP) was performed. The results of immunoprecipitation of RAD18 (αRAD18) or ubiquitylated RAD18 (αFK2) were detected on immunoblots. (D) HeLa cells stably expressing YFP-RAD18 or transiently expressing YFP-mutant RAD18, and wild-type HeLa cells were transfected with siRNA targeting endogenous RAD18 (si-endoRAD18). Forty-eight hours later, cells were lysed (input) and immunoprecipitation with YFP antibody (IP αYFP) was performed. The expression levels of RAD18 and HR6A/B in the lysate, detected with αRAD18 and αHR6A/B, are shown as input. (E) Quantification of the amount of co-immunoprecipitated HR6A/B in the IP results of (D). The amount of HR6A/B that co-precipitated with wild-type RAD18 was set at 1.0. The error bars in the graph represent the standard deviation of three independent experiments.

RAD18 contains several distinct functional domains; a C3HC4 type RING finger, a C2HC type Zinc finger, a SAP domain, and a HR6A/B binding domain (HR6BD) (Figure 1A) [14]. We generated several mutations in human RAD18 tagged with YFP at the N-terminus, as shown in Figure 1A.

In the analysis of the immunoblots, HeLa cells stably expressing YFP-RAD18 at a level similar to endogenous RAD18 (Figure 1B) [28] were used as a control. It has been shown that RAD18 is monoubiquitylated [2], [14] in a HR6A/B-dependent manner [14]. In agreement with these findings, both endogenous RAD18, and overexpressed wild-type YFP-RAD18 appeared as a double band separated by 7 kDa on immunoblots (Figure 1B), and the upper band was recognized by an antibody against conjugated ubiquitin (FK2) (Figure 1C). In the experiments presented in Figure 1D, endogenous RAD18 was transiently downregulated by siRNAs in HeLa cells as previously described [28]. Transient or stable knockdown of RAD18 is very efficient, with more than 90% downregulation of protein expression [28] (Figure S1A–C). For YFP-RAD18, a RAD18 double band was still formed for both RING finger mutants (Figure 1D, input), but not for the Zinc finger or HR6BD mutants, that were expressed as a single unmodified band, based on the size in comparison to the wild type protein (Figure 1D). These findings suggest that the ubiquitylation of RAD18 is independent of the RING domain, but does depend on the Zinc finger and the HR6BD. A mutant carrying mutations in all putative autoubiquitylation sites (K161R/K261R/K309R/K318R) (Δubi) [14] still appeared as a doublet that was identical to the doublet observed for wild type RAD18 (Figure S1D). In addition, based upon evolutionary conservation of lysines between species, we induced single mutations at 4 additional candidate lysines (K197R, K230R, K241R, and K245R) that might represent autoubiquitylation sites. The results suggest that none of these sites is required for RAD18 ubiquitylation (Figure S1E), indicating the presence of another unknown putative ubiquitylation site in RAD18 that is required for mono-ubiquitylation *in vivo*. However, it cannot be excluded that the autoubiquitylation site changes when the preferred lysine is mutated.

### Binding of HR6A/B to RAD18 requires not only an intact RING finger and HR6A/B binding domain, but also the SAP domain *in vivo*

In *Saccharomyces cerevisiae*, Rad6 binds to Rad18 via the C-terminal domain of Rad18, as shown in a yeast two-hybrid assay [13]. In addition to the C-terminal domain, the RING finger seems to be also essential for binding of mammalian RAD18 to HR6A/B, as found in an *in vitro* assay [14] and yeast two-hybrid assay [55]. In the present study, we performed pull-down assays using HeLa cells expressing YFP-tagged RAD18 carrying different mutations, while endogenous RAD18 was downregulated by siRNAs (Figure 1D), and analyzed the interaction between RAD18 and HR6A/B (Figure 1E). A 40-fold reduction in the amount of co-precipitated HR6A/B was observed for the RAD18 A357R mutant (HR6BD), compared with wild-type. In addition, the RING finger mutants also showed a 5–10 fold decreased efficiency of HR6A/B co-precipitation (Figure 1D and 1E), suggesting that both the RING finger and HR6BD mediate interaction with HR6A/B *in vivo*. Indeed, a double mutant RAD18 (C28F/A357R) completely failed to co-precipitate HR6A/B, confirming that HR6A/B interacts with RAD18 via both the HR6BD and the RING finger domains *in vivo* (Figure 1D and 1E). Surprisingly, the SAP domain mutants also showed a reduced co-precipitation efficiency for HR6A/B, whereas the Zinc finger mutants and Δubi mutant behaved similar to the wild type protein with respect to co-immunoprecipitation of HR6A/B (Figure 1D and 1E, Figure S1D for Δubi).

To analyze the E3 ligase function of RAD18, we examined the ability of RAD18 mutants to ubiquitylate PCNA, which is a well known substrate of RAD18 [10], [56]. In accordance with the findings of Huang et al. (2009) [1], mutations in the SAP domain precluded PCNA ubiquitylation as well as mutations in the RING finger or the HR6BD (Figure S1F). Together with our data obtained for co-immunoprecipitation of RAD18 mutants with HR6A/B, this suggests that interaction of HR6A/B with RAD18 is crucial for PCNA ubiquitylation, since the level of PCNA ubiquitylation correlated with the amount of HR6A/B that co-precipitated with the different YFP-RAD18 mutants.

Nakajima et al. (2006) [57] and Huang et al. (2009) [1], previously showed that the accumulation, of RAD18 on chromatin surrounding sites of DNA damage depends on the Zinc finger. Here we studied which domains of RAD18 are required for the cell cycle regulated pattern of nuclear RAD18 accumulation in the absence and presence of exogenous DNA damage. We also used time lapse analyses to follow the localization of the Zinc finger mutant of RAD18 throughout the cell cycle in the absence of exogenous damage. Our data confirm and extend the previous reports. We found that accumulation of RAD18 in foci throughout the cell cycle, and in the nucleolus in late G2 all depends on its Zinc finger, and is independent of its RING finger domain, the SAP domain, and the HR6BD (Figure S2, Movie S1 for wild type and Movies S2 and S3 for Zinc finger mutant D221A).

### RAD18 accumulation at DSBs depends on RPA

Having established that the Zinc finger domain of RAD18 is required for the formation of RAD18 foci throughout the cell cycle and upon irradiation, we next investigated which proteins are required for RAD18 recruitment to chromatin.

**Figure 2:**
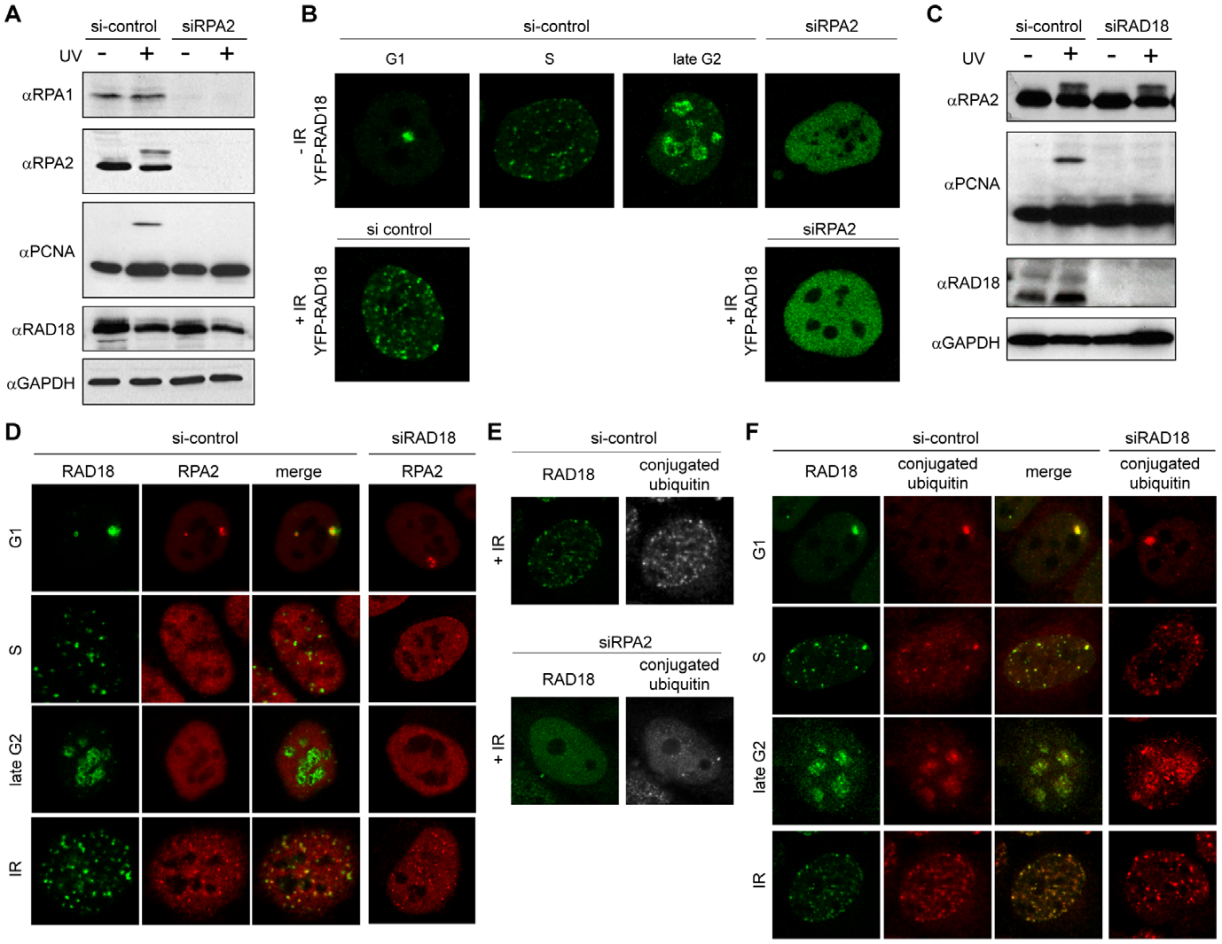
RPA2-dependent RAD18 accumulation at sites of DNA damage. HeLa cells stably expressing YFP-RAD18 were transfected with siRNA against RPA2 (siRPA2) (A, B, E), or RAD18 (siRAD18) (C, D, F) for 48 hours. Non-targeting siRNA (si-control) was used as a control. (A, C) Cells were irradiated with 20 J/m^2^ UV-C and whole-cell extracts were prepared 8 h later. Expression levels of RPA1, RPA2, mono-ubiquitylated PCNA, and RAD18 were analyzed with immunoblots using different antibodies as indicated. GAPDH was used as a loading control. (B) Confocal images of living YFP-RAD18 HeLa cells in all cell cycle phases as indicated above the pictures. For cells treated with siRPA2, it was not possible to determine the cell cycle phase. Subsequently, cells were exposed to IR (5 Gy), and living cell nuclear images were captured 30 min after IR. (D, E, F) Cells were exposed to IR (5 Gy), and fixed 30 min later. Antibodies recognizing RPA and RAD18 were used to visualize the proteins as indicated. Anti-FK2 was used to visualize conjugated ubiquitins. Cell cycle phases are indicated on the left of each panel.

In order to examine whether RPA is required for RAD18 accumulation at sites of γ-irradiation-induced DNA damage, RPA2 was depleted using siRNA, and foci formation of RAD18 was analyzed. Downregulation of RPA2 expression (Figure 2A) also reduced the expression level of RPA1, and no PCNA mono-ubiquitylation was detected following UV irradiation. It needs to be taken into account that RPA-deficient cells are very likely blocked in G1 phase, since RPA is essential for DNA replication. In these RPA-deficient cells, we were no longer able to detect spontaneous or IR-induced foci formation of RAD18 (Figure 2B), although the expression level of RAD18 did not change (Figure 2A). Note that RAD18 is able to form IR-induced foci in G1 phase of wild type cells [28]. Since downregulation of RAD18 did not influence either RPA expression (Figure 2C) or localization (Figure 2D), RPA most likely acts upstream of RAD18 at IR-induced DSBs. RPA and RAD18 have been shown to interact directly in yeast [26], and therefore, RPA may directly recruit RAD18 to the site of DNA damage. However, RPA shows a focal accumulation at damage sites, whereas RAD18 accumulation is more diffuse [28] (Figure 2D). In addition, the two proteins do not always colocalize (Figure 2D), indicating that RPA is most likely not directly involved in recruiting RAD18 to the chromatin that surrounds the DSB site. RPA probably functions not only in the actual physical repair of the DNA, but also in the DNA damage response pathway. Surprisingly, we found that accumulation of conjugated ubiquitins to chromatin surrounding DSBs, a known early step in the DNA damage response [33], was also RPA dependent (Figure 2E). Together with the results from the mutation analyses, this prompted us to examine the cell-cycle-dependent localization pattern of conjugated ubiquitins in comparison with that of RAD18. We found an overall colocalization of RAD18 with conjugated ubiquitins at all cell cycle phases, and after IR (Figure 2F). This localization pattern of conjugated ubiquitins was independent of RAD18 expression (Figure 2F). These observations suggest that the localization of RAD18 to chromatin in the absence of exogenous damage is preceded by ubiquitylation of an unknown chromatin component.

### RAD18 binds to ubiquitylated chromatin components including histone H2A

**Figure 3:**
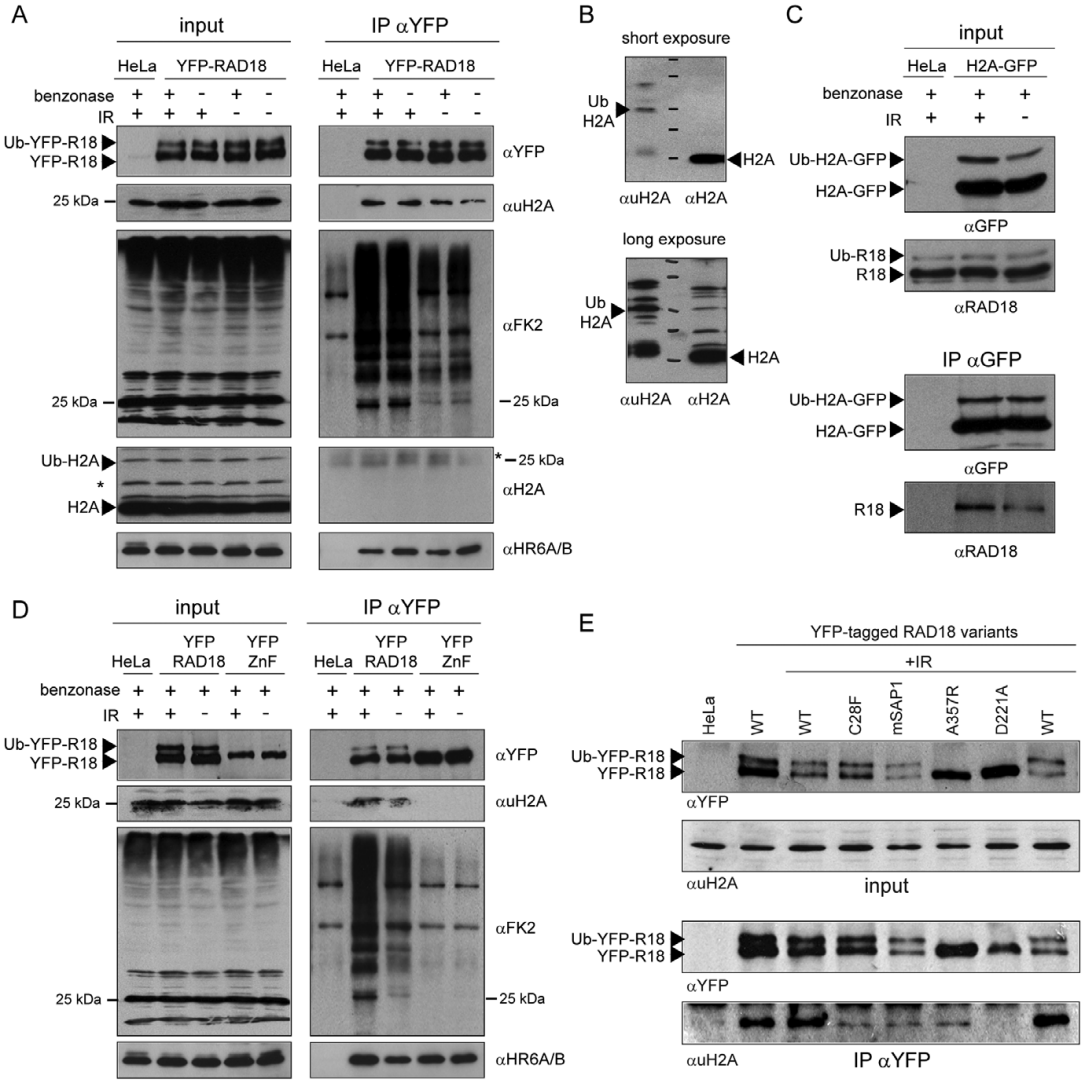
Interaction of RAD18 with ubiquitylated histone H2A via its Zinc finger following IR. (A) HeLa cells stably expressing YFP-RAD18 were exposed to IR (10 Gy). One hour later, cells were lysed and immunoprecipitation (IP) was performed using an YFP antibody. Subsequently, co-precipitation of ubiquitylated histone H2A (uH2A), conjugated ubiquitin (FK2), histone H2A, and HR6A/B was detected on immunoblots using antibodies as indicated. The expression level of the proteins in the input samples is shown in the left panel. Wild-type HeLa cells were used as a control. The asterisk indicates a nonspecific band (light chain of IgG in IP αYFP, αH2A). (B) Expression level of H2A and ubiquitylated H2A was analysed in HeLa cells using two different antibodies as shown in the figures. (C) Under the same experimental conditions as in (A), HeLa cells stably expressing H2A-GFP were analyzed for the interaction of GFP-H2A with endogenous RAD18. Input samples are shown in the top panels. In the bottom panels (IP αGFP), (co) immunoprecipitation of (ubiquitylated) H2A and RAD18 are shown as indicated. (D) Wild type or Zinc finger mutant (D221A;YFP-ZnF) YFP-RAD18 were expressed in HeLa cells in which endogenous RAD18 was stably downregulated (see Figure S1B and S1C). Under the same experimental condition as in (A), interaction of either YFP-RAD18 or YFP-ZnF with ubiquitylated H2A was analyzed by immunoprecipitation. Subsequently, co-precipitation of uH2A, conjugated ubiquitin, and HR6A/B was detected on immunoblots using antibodies as indicated. The expression level of the proteins in the input samples is shown in the left panel. Wild-type HeLa cells were used as a control. (E) Under the same experimental conditions as in (B) but without benzonase treatment, several RAD18 mutants as indicated were analyzed for their interaction with ubiquitylated H2A.

Following induction of DSBs, the surrounding chromatin at the break sites undergoes various modifications. One of the first modifications is phosphorylation of histone H2A variant H2AX (γH2AX) by ATM (serine/threonine protein kinase ataxia telangiectasia mutated) [32]. MDC1, a mediator protein, is immediately recruited to sites of DSBs, interacts directly with γH2AX via its BRCT domains [58], and plays a crucial role in the DNA damage response pathway [40], [59]. Subsequently, histone H2A and H2AX are ubiquitylated in the chromatin surrounding DSBs by the E3 ubiquitin ligases RNF8 and RNF168 in a MDC1-dependent manner [33], [34], [35]. Since RAD18 carrying a mutation in its ubiquitin-binding Zinc finger did not form any DSB-associated foci (Figure S2, and Movies S2 and S3), we hypothesized that RAD18 is recruited to the chromatin surrounding a DSB through binding to ubiquitylated H2A and/or H2AX. This idea is supported by the recent finding that the accumulation of RAD18 at sites of DSBs is dependent on RNF8 [1]. To investigate this hypothesis, we examined the physical interaction of wild-type RAD18 with ubiquitylated H2A by an immunoprecipitation assay in HeLa cells with and without IR exposure. Cell lysates were pretreated with nuclease (Figure S1G), to exclude that any co-immunoprecipitation observed resulted from indirect interactions via DNA. On immunoblots, we did not observe a clear increase in the amount of either ubiquitylated H2A or conjugated ubiquitin recognized by the FK2 antibody in cells treated with IR compared to controls (Figure 3A and 3D, input). This might be due to the abundant amounts of ubiquitylated H2A that localize to silenced genomic regions, independent of DNA damage, and has also been observed by others [60]. Upon immunoprecipitation of YFP-RAD18, we could clearly co-immunoprecipitate ubiquitylated H2A (Figure 3A, IP αYFP), and the amount was somewhat increased after IR (Figure 3A, IP αYFP). This suggests that RAD18 is recruited to DSB repair sites at least in part via interaction with mono-ubiquitylated H2A. Non-ubiquitylated H2A was not specifically co-precipitated (Figure 3A, IP αYFP). The H2A antibody detected ubiquitylated H2A only upon overexposure in the input fraction (Figure 3B), and not in the IP, most likely because the low molecular weight of IgG band co-migrated with the ubiquitylated H2A band (Figure 3A, IP αYFP). Using a HeLa cell line that overexpresses H2A-GFP [61], [62], we detected increased ubiquitylation upon irradiation (Figure 3C, input), as well as increased co-immunoprecipitation of endogenous RAD18 (Figure 3C, IP αGFP). Intriguingly, ubiquitylated H2A-GFP interacted only with the non-ubiquitylated form of RAD18 (Figure 3C, IP αGFP). In order to analyse whether RAD18 interacts with ubiquitylated H2A through the Zinc finger of RAD18, we performed co-immunoprecipitation assays using HeLa cells in which endogenous RAD18 was stably downregulated via shRNA expression (see Material and Methods and Figure S1B and S1C), and that transiently expressed either wild-type or Zinc finger mutant YFP-RAD18 (carrying 5 silent mutations to prevent downregulation by the shRNA, Figure 3D). Ubiquitylated H2A was only co-precipitated by wild type YFP-RAD18 and not by the RAD18 Zinc finger mutant D221A, and the amount of co-precipitated ubiquitylated H2A was increased in cells treated with IR (Figure 3D, IP αYFP). Another RAD18 Zinc finger mutant (C207F) also did not show any interaction with ubiquitylated H2A (data not shown). In addition to the interaction of RAD18 with ubiquitylated H2A, our immunoprecipitation results also indicate that RAD18 interacts with many additional ubiquitylated proteins, in a Zinc finger-dependent way, making it highly likely that apart from H2A and H2AX, other ubiquitylated chromatin components will also be used by RAD18 to bind to chromatin near DSB repair sites. Note that these unknown ubiquitylated proteins which interact with RAD18 show distinct banding patterns on immunoblots compared to the input materials, indicating that RAD18 does not randomly bind to any ubiquitylated protein (Figure 3A). As a control for the assay, co-immunoprecipitation of HR6A/B was checked, and both wild-type RAD18 and the Zinc finger mutant were able to co-precipitate HR6A/B (Figure 3D, IP αYFP). RAD18 carrying mutations in either the RING finger, SAP domain, or HR6BD was still capable to interact with ubiquitylated H2A, although these RAD18 mutants showed considerably reduced amounts of co-precipitated ubiquitylated H2A (Figure 3E).

### RAD18 always colocalizes with ubiquitylated H2A during the cell cycle in HeLa cells, but rarely with ubiquitylated H2A at the Barr body in human female fibroblasts

**Figure 4:**
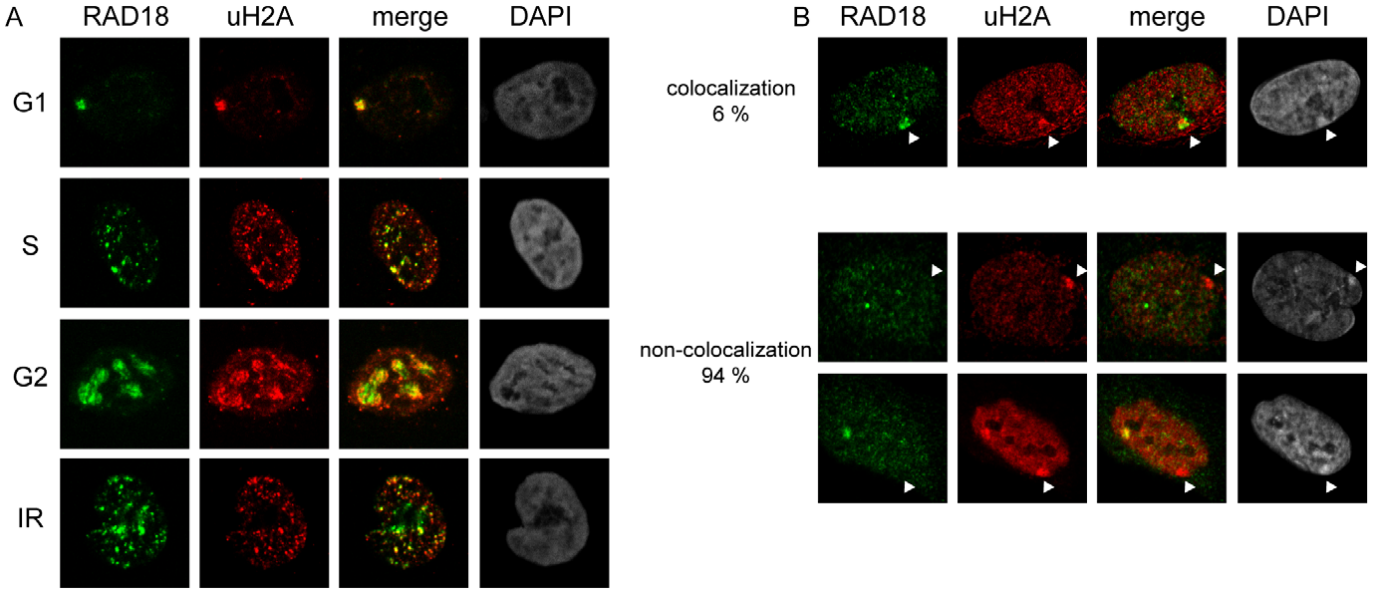
RAD18 always colocalizes with uH2A in HeLa cells, but rarely with the uH2A-enriched Barr body. (A) Localization of RAD18 and uH2A was visualized using the indicated antibodies. Cell phases were determined by the subnuclear distribution pattern of RAD18. To induce DSBs, HeLa cells were exposed to IR (5 Gy) and fixed 30 min later. Cell cycle phases are indicated on the left of the pictures. (B) Localization of RAD18 and uH2A in human primary female fibroblast cells. Arrowheads indicate the Barr body, based on the intense DAPI staining.

The immunocytochemical analyses of conjugated ubiquitin in relation to the localization of RAD18 in HeLa cells indicate that RAD18 localization to chromatin is tightly correlated to the presence of ubiquitylated H2A and other ubiquitylated chromatin components (Figure 2F). To examine this further, we studied the localization of RAD18 and ubiquitylated H2A during the cell cycle and after IR in HeLa cells (Figure 4A). Indeed, similar to our findings with the FK2 antibody, RAD18 and ubiquitylated H2A showed colocalization (Figure 4A). These results confirm that ubiquitylated H2A is one of the main ubiquitylated chromatin components.

Next, to analyze whether RAD18 always colocalizes with regions that show enhanced H2A ubiquitylation, we analyzed human female primary fibroblast cultures. In these cells, dosage compensation has resulted in the inactivation of one of the two X chromosomes, and the inactivated chromosome, forming the Barr body, is known to be enriched for ubiquitylated H2A and trimethylation of histone H3 at lysine 27 (H3K27me3). Immunocytochemical analyses of ubiquitylated H2A, conjugated ubiquitins, H3K27me3, and RAD18 signals in these cells revealed that the Barr body, visible by dense DAPI staining, and H3K27me3 (data not shown) was mostly not enriched for RAD18, even if uH2A staining was enhanced in this region. In a small percentage of the nuclei (6%), RAD18 enrichment was detected at the Barr body (Figure 4B). Sometimes, the cells displayed accumulation of uH2A in two separated areas; one representing the Barr body, and the other positive for RAD18 (Figure 4B, the lowest panels). At the Barr body, it is not known whether H2A is only mono-ubiquitylated, or whether perhaps also poly-ubiquitylation occurs. The immunoprecipitation analyses indicate that RAD18 interacts with mono-ubiquitylated H2A, but it may also interact with some polyubiquitylated forms of H2A. Thus it is not clear why RAD18 only rarely colocalizes with uH2A staining in this region. It might be suggested that the interaction between RAD18 and uH2A depends on the context; i.e. the presence or absence of specific additional chromatin-associated factors.

### RAD18-dependent accumulation of RAD9 at damaged sites

**Figure 5:**
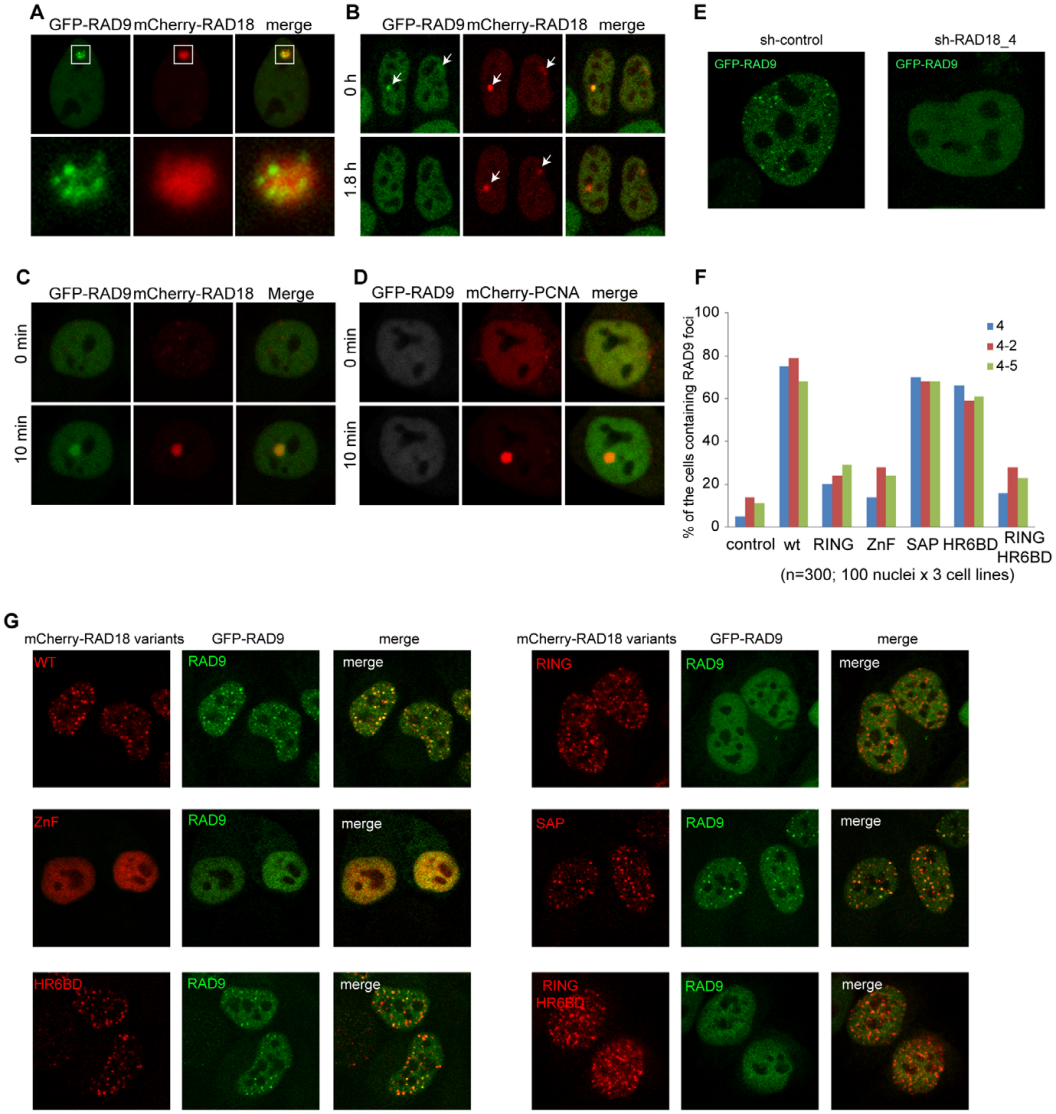
RAD18 facilitates RAD9 localization at induced damaged sites. (A) A confocal image of a living HeLa cell expressing GFP-RAD9 and mCherry-RAD18 in G1 phase. The enlarged G1 foci are shown in the lower panels. (B) Images from time-lapse analysis of living HeLa cells expressing GFP-RAD9 and mCherry-RAD18 in G1 phase. The first moment at which RAD18 was found to colocalize with RAD9 was set at time 0. Arrows in the pictures indicate the G1 foci. (C) HeLa cells expressing GFP-RAD9 and mCherry-RAD18 were locally irradiated with a multi-photon laser (MPL) at 75 mW. (D) The expression of RAD18 was downregulated with siRNA against RAD18 (si-RAD18) in HeLa cells stably expressing GFP-RAD9 and transiently co-expressing mCherry-PCNA. Subsequently, the cells were locally irradiated with MPL at 75 mW. As a control, mCherry-PCNA was also analyzed. (E) GFP-RAD9 was overexpressed in HeLa cells stably expressing either shRNA against RAD18 or non-targeting shRNA. Subsequently, cells were irradiated with IR (5 Gy) and confocal images of living cells were captured after 2 hours. (F) mCherry-tagged RAD18 variants and GFP-RAD9 were co-expressed in RAD18 knockdown cell lines. Subsequently, cells were irradiated with IR (5 Gy) and fixed after 2 hours. Nuclei expressing both mCherry-RAD18 variants and GFP-RAD9 were analyzed and the number of nuclei containing IR-induced RAD9 foci is shown in the graph. 100 nuclei in each mutant and in each *RAD18* knockdown cell line were counted. (G) Representative pictures of nuclei expressing both mCherry-RAD18 variants and GFP-RAD9 examined in (F) are shown.

Next, we attempted to analyze the downstream effects of RAD18 localization at DSB repair sites. In eukaryotes, RAD9, RAD1 and HUS1 form the PCNA-like heterotrimeric 9-1-1 checkpoint complex, which plays a crucial role in cell cycle checkpoint signaling following endogenous and exogenous DNA damage [63], [64]. The 9-1-1 complex is known to be activated by replication fork stalling and to initiate the checkpoint signaling cascade during S phase [22], whereas RAD18 facilitates the RDB pathway at stalled replication sites [10], [56]. It has been shown that both RAD18 and the 9-1-1 clamp loader directly interact with the RPA complex [25], [65], suggesting a functional interaction at stalled replication sites. One of the components of the 9-1-1 complex, RAD9, is known to associate with chromatin at sites of DSBs in human cells [49], [66]. Therefore we investigated the possible functional interaction between RAD9 and RAD18 in mammalian cells. First, their dynamic localization was examined by time-lapse experiments in living cells. In G1 phase, RAD18 forms a few foci which also contain RAD51, RPA and γH2AX [28]. In these so-called G1 foci, we observed RAD9 accumulation (Figure 5A). The higher magnification in the lower panels of Figure 5A shows that RAD9 accumulates as multiple foci, whereas RAD18 shows a more diffuse accumulation, surrounding RAD9 foci. This pattern of RAD9 localization in G1 is very similar to what has been observed for RPA and RAD51 [28]. The localization of RAD9 foci within the RAD18-positive chromatin area in G1 was found for only a few hours (1–2 h). Subsequently, RAD9 foci disappeared (Figure 5B), although the RAD18 foci remained present until the cell entered S phase [28]. In S and G2 phases, RAD9 showed a homogeneous distribution in the nucleus and did not form any foci. In contrast, RAD18 accumulates in many foci in S and G2 phases [28]. To further analyze the functional relation between RAD18 and RAD9 at damaged sites, local damage was induced with a multi-photon laser (MPL) [28]. This revealed that both RAD18 and RAD9 accumulated at the damaged sites (Figure 5C). Interestingly, using HeLa cells in which RAD18 was transiently downregulated with siRNA (Figure S1A) or stably downregulated with shRNA (Figure S1B and S1C), RAD9 no longer accumulated at damaged sites following MPL (Figure 5D) or IR exposure (Figure 5E). To analyze the ability of different RAD18 mutants to recruit RAD9 following IR, wild type and mutant RAD18 (mutation in either the RING finger, Zinc finger, SAP domain, or HR6BD, and a double mutant in the RING finger and HR6BD) were transiently co-expressed with GFP-RAD9 in three different *RAD18* knockdown HeLa cell lines (Figure 5F and 5G). Wild type RAD18 was able to rescue foci formation of RAD9 after IR in approximately 80% of the cells (Figure 5F and 5G). Expression of RAD18 containing the SAP domain mutation (mSAP1) or a mutation in the HR6BD (A357R) also rescued RAD9 accumulation, RAD18 carrying a point mutation in the RING finger (C28F), or the Zinc finger (D221A), and the double mutant in the RING finger and the HR6BD (C28F/A357R) showed severely reduced IR-induced RAD9 accumulation compared to the wild type (Figure 5F and 5G). In control experiments, we observed RAD9 accumulation in approximately 5–10% of *RAD18* knockdown cells (Figure 5F), in accordance with a more than 90% downregulation of RAD18 expression in the *RAD18* knockdown cell lines (Figure S1B). In addition, *RAD18* knockdown HeLa cells expressing RAD18 mutants used in this experiment showed an equivalent cell growth rate and time span of the cell cycle compared to *RAD18* knockdown HeLa cells expressing wild-type RAD18 (data not shown). Taken together, these data suggest that recruitment of RAD9 to IR-induced DSBs requires the Zinc finger domain of RAD18 and its E3 ligase activity. To analyze this further, we studied physical interactions between RAD18 and components of the 9-1-1 complex by the yeast two-hybrid assay in the absence and presence of DNA damage (HU, CPT, MMS, and IR). In accordance with recent data concerning yeast RAD18 and the 9-1-1 complex [54], no direct interaction between human RAD18 and the 9-1-1 complex was detected (Figure S3A), although interactions among the 9-1-1 complex components (Figure S3B), between RAD18 and HR6A/B, and also between RAD18 and PCNA (Figure S3C) were detected. These results suggest that RAD18 might not interact stably or directly with the 9-1-1 complex. Next, we investigated whether knockdown of RAD18 and the subsequent lack of foci formation of RAD9 upon IR also affected activation of the downstream checkpoint kinases CHK1 and CHK2, but the response to 10 Gy IR was identical in control and *Rad18* knockdown cells (Figure S4).

## Discussion

### RPA is required for RAD18 and ubiquitin accumulation at chromatin surrounding DSBs

Two distinct manners of recruitment of RAD18 to sites of DNA damage can be observed. First, RAD18 has a direct DNA binding ability mediated by the SAP domain, and/or RPA bound to ssDNA mediates recruitment of RAD18 to the ssDNA. RPA and the SAP domain of RAD18 are both required for PCNA ubiquitylation in the RDB pathway [25]. Second, recruitment of RAD18 to the chromatin surrounding DSB sites depends on the ubiquitin-binding Zinc finger domain of RAD18. Surprisingly, this type of RAD18 recruitment also requires RPA, but this must occur indirectly, since RAD18 shows a more diffuse localization compared to RPA at DSB sites, and RPA and RAD18 show relatively little colocalization during the cell cycle.

RPA is a single-stranded DNA (ssDNA) binding protein, essential for both DNA replication and recombination [18], [19], [20], [21], [22], [23]. According to current models [25], [26], stalled replication forks expose ssDNA covered by RPA which is required for PCNA ubiquitylation [25], [27] and the recruitment of RAD18 [25], [26]. Once RAD18 is bound to RPA, or to ssDNA, or to both, it may ubiquitylate PCNA. RAD18 (complexed with HR6A/B) carrying mutations in the Zinc finger most likely is still capable of binding to ssDNA via the SAP domain, and via interaction with RPA, and could be present at stalled replication forks, perhaps undetectable due to the small number of molecules present, below the threshold for foci formation. Since the interaction between RAD18 and HR6A/B partially depends on an intact SAP domain, we were unable to determine whether the SAP domain functions in PCNA ubiquitylation by mediating RAD18 binding to DNA *in vivo*, or as a third domain required for RAD18-HR6A/B interaction, or both. Our observations on the differential requirement of the Zinc finger and the SAP domain in mediating PCNA ubiquitylation nicely mirror the results described by Nakajima et al. (2006) who found that the Zinc finger domain of RAD18 is required for focal localization of RAD18 at damaged sites in cells irradiated with an UVA or UVC laser. In addition, RAD18 carrying a mutation in the Zinc finger domain could restore Polη foci formation at sites of stalled replication, whereas a SAP domain mutant could not [57].

Our observation that not only the accumulation of RAD18, but also that of ubiquitylated chromatin components depends on RPA is surprising. Ubiquitylation of chromatin in response to DSBs requires the ubiquitin ligases RNF8 and RNF168 [33], [34], [37], and their accumulation in turn depends on MDC1 which is associated with γH2AX [33], [37]. In RPA-depleted cells, γH2AX still accumulates at DSBs, and therefore it would be expected that MDC1 and RNF8/RNF168 would also be recruited. However, apart from γH2AX and MDC1, specific chromatin remodelling is also required to recruit RNF8 [67]. The absence of FK2 staining in irradiated *RPA* knockdown cells indicates that RPA accumulation and function are somehow linked to this branch of the DDR pathway.

### RAD18 is recruited to sites of DSB-repair via binding to ubiquitylated H2A and other ubiquitylated chromatin components

In the present study, we found a physical and specific interaction between RAD18 and ubiquitylated H2A via its Zinc finger. In addition, certain other ubiquitylated chromatin components may also be bound by RAD18. The distribution pattern of conjugated ubiquitin on chromatin in the nucleus during the cell cycle was identical to that of RAD18, indicating that chromatin association of RAD18 primarily occurs through ubiquitin-binding. Intriguingly, immunocytochemical analyses in human female primary fibroblast cells revealed that the Barr body, known to be marked with uH2A, was mostly not enriched for RAD18, even if uH2A and FK2 staining was enhanced in this region. These data indicate that the interaction of RAD18 with ubiquitylated chromatin components including uH2A is related to DNA damage, and possibly regulated. Such regulation might occur via phosphorylation of RAD18 by ATM kinase, similar to what occurs for many other proteins involved in the DNA damage response pathway.

### RAD18 ubiquitylation depends on the Zinc finger domain, but does not require the RING finger domain

*In vitro* ubiquitylation of RAD18 depends on HR6A/B [2]. In addition, the RAD18 Zinc finger is essential for mono-ubiquitylation of RAD18 *in vivo* (Fig. 1D and [2]). In *RNF8-*knockdown cells, in which RAD18 is no longer recruited to DSB repair sites, RAD18 ubiquitylation is still observed [1], indicating that it is unlikely that it is the aberrant localization of the Zinc finger mutant that causes the lack of ubiquitylation of RAD18. Surprisingly, we find that the RING finger of RAD18 is not required for RAD18 ubiquitylation. Previous data indicated that the RING finger might be required for RAD18 autoubiquitylation, but the amount of mutant protein that was expressed was too low to draw a definite conclusion [2]. It might be suggested that ubiquitylation of RAD18 is performed by a different E3 ligase, but this would then also depend on interaction of RAD18 with HR6A/B. *In vitro* ubiquitylation of RAD18 by HR6A/B does not require an additional E3 [2]. However, it should be noted that these *in vitro* conditions involved an extremely high HR6A/B concentration. Recently, it was shown that a dimer of full length RAD18 binds only a single HR6A/B molecule *in vivo* [68], and that binding of the HR6BD of RAD18 to HR6A/B competes with noncovalent binding of HR6A/B to ubiquitin, thereby preventing polyubiquitylation of the target [69]. The observed *in vivo* polyubiquitylation of RAD18 in the presence of proteasome inhibitors [2] therefore suggests that it cannot be excluded that RAD18 is ubiquitylated by another E3 enzyme, that may or may not act together with HR6A/B, but whose activity somehow depends on the HR6BD of RAD18. Alternatively, the residual interaction between RAD18 and HR6A/B through the HR6BD domain in the RING finger mutant may still allow RAD18 ubiquitylation, independent of E3 activity, in a mechanism described by Hoeller et al. (2007) [70] for ubiquitin binding domain (UBD) containing proteins. In this mechanism, the UBD can directly cooperate with the Ub-charged E2 enzyme, and allow ubiquitylation of the host protein, in the absence of an E3 ligase [70]. In RAD18, the Zinc finger represents a UBD [14]. Thus, interaction of RAD18 with Ub-charged HR6A/B, via the HR6BD and the Zinc finger UBD, might allow RAD18 ubiquitylation even if the E3-ligase function of RAD18 is absent. In accordance with this notion, RAD18 mutants that are unable to bind HR6A/B are not ubiquitylated. This E3-independent ubiquitylation of UBD-containing proteins is thought to lead to their inactivation due to intra- or inter-molecular association of the covalently attached ubiquitin moiety with the UBD (reviewed in [71]). This would fit with the idea that mono-ubiquitylation of RAD18 occurs when it is not functionally engaged in the ubiquitylation of a substrate, for example when it is freely diffusing in the nucleoplasm or cytoplasm. It is also in accordance with our observation that precipitation of (ubiquitylated) H2A co-precipitates only the non-ubiquitylated form of RAD18.

### RAD18 facilitates RAD9 recruitment selectively to G1 foci and DSB repair sites

In the present study, we showed that human RAD9 localizes at IR-induced damaged sites in a RAD18-dependent manner. This function depends on the Zinc finger and ubiquitin ligase activity of RAD18. In accordance with our findings, Huang et al. (2009), also showed that the role of RAD18 in HR requires the Zinc finger and RING finger domains of RAD18, although their data indicated that the E3 ligase activity of RAD18 is not required to promote HR [1]. In contrast, two different groups recently showed that the ubiquitin ligase activity of RAD18 is required for Fanconi Anemia pathway activation at DSBs, independent of PCNA ubiquitylation [72], [73]. Thus, RAD18 plays several roles at sites of DSB repair, some of which are dependent, and others are independent from its E3 ligase activity. We have not detected a direct stable interaction between RAD18 and the 9-1-1 complex (Figure S3A), and could not obtain evidence for RAD18-dependent ubiquitylation of the 9-1-1 complex (data not shown). It is somewhat surprising that the HR6BD is not required for RAD9 recruitment to DSBs. We suggest that in the absence of the HR6BD, the RING finger could still be sufficient to maintain the RAD18-HR6A/B interaction required for ubiquitylation of an unknown substrate which leads to RAD9 recruitment.

Earlier reports have also shown association of human RAD9 with chromatin after DNA damage [50], and depletion of mammalian RAD9 leads to IR sensitivity [46], [51], [52] in particular in the S and G2 phase of the cell cycle [52]. Loading of the 9-1-1 complex to damaged sites depends on the mammalian clamp loader RAD17, but occurs independent of ATM/ATR localization [74]. One of the known functions of the 9-1-1 complex is to activate CHK1, a kinase which is involved in the checkpoint signaling pathway [45], [46], [47], [48]. In addition, RAD9 most likely performs a direct role in homologous recombination repair following IR [46], [49], [52]. Interestingly, RAD9 has also been shown to be required for full activation of FANCD2 at interstrand cross-links and following IR [75]. We have shown in the present study that depletion of RAD18, and thereby of RAD9 from the damaged site, does not cause any deficiency in phosphorylation of CHK1 or CHK2, also suggesting a role for RAD18 and RAD9 at IR-induced DNA damage, independent from the function of cell cycle checkpoint control by the 9-1-1 complex. The localization pattern of RAD9 within the RAD18-positive area in G1 shows a striking resemblance to the RPA and RAD51 pattern, indicating that RAD9 localizes to the G1 foci that are attempting to perform HR, where it may interact with RPA [65], [76] and/or RAD51 [52]. It might be suggested that the ligase activity of RAD18 is required, together with RAD9, to activate the Fanconi Anemia pathway at DSB repair sites. In addition, a ligase-independent function of RAD18 may involve interaction with RAD51C and/or 53BP1 to directly stimulate HR or NHEJ, depending on the context.

### Conclusion

Taken together, our data suggest that RPA is required for RAD18 localisation at DSBs. This most likely occurs through indirect recruitment of RNF8/RNF168 to DSBs, and subsequent ubiquitylation of H2A and other chromatin components. The RAD18 zinc finger domain binds to these ubiquitin moieties, resulting in efficient RAD18 recruitment, and this facilitates RAD9 accumulation via the ubiquitin ligase activity of RAD18. The role of RAD18 in stimulating HR may thus be mediated in part through direct actions of RAD9 interacting with RAD51 and/or RPA.

## Materials and Methods

### DNA constructs, transfection and cell culture

Design of the YFP-RAD18 construct containing four silent mutations, and generation of the HeLa cell line stably expressing YFP-RAD18 were described elsewhere [28]. All mutant RAD18 constructs (see below) were generated by PCR reactions (30 sec at 90°C, 1 min at 55°C, and 12 min at 68°C for 25 cycles) with primers carrying mutations (Table S1). The YFP-RAD18 construct was used as template DNA. 50 µl of the PCR products were mixed with 2 µl of *Dpn*I (Bio England) for 2 hours at 37°C. XL1-blue (Strategene) was transformed with 2 µl of the *Dpn*I digested PCR products. Plasmids containing appropriate mutants were digested with *Eco*RI and *Bam*HI and sub-cloned into pEYFP-C1 (Clontech) and mCherry-C1 (Clontech). pEGFP-RAD9 construct was kindly provided by Daniel Warmerdam [77].

For yeast two-hybrid assays, open reading frames of human *RAD18*, *PCNA*, *RAD9*, *HUS1* and *RAD1*, and mouse *Hr6a* and *Hr6b* genes were amplified using primer sets (Table S2) from cDNA and sub-cloned into pGADT7 and pGBKT7 plasmids (Clontech).

To stably downregulate RAD18, two complementary nucleotides (Table S3) containing a hairpin loop structure which targets endogenous *RAD18* (shRAD18) were annealed (1 min at 94°C, 4 min at 65°C, 5 min at 30°C and 2 h at 4°C) and sub-cloned into pSuper-puro vector (OligoEngine). To generate HeLa cells stably expressing pSuper-shRAD18, cells were transfected with 2 µg of the pSuper-shRAD18 plasmid using FuGene6 (Roche). Cells were cultured for 7 days after transfection with medium containing 0.1 mM of puromycine. Surviving colonies were selected, and RT-PCR and immunoblot analysis were used to further select cell lines that showed downregulation of RAD18. As a control, the empty pSuper-puro vector was transfected followed by the same selection procedure. In order to transfect mutant RAD18 in the *RAD18* knockdown cell line, five silent mutations were introduced in the *RAD18* gene in the region that is targeted by pSuper-shRAD18 (Table S1), and this construct was subcloned into pEYFP-C1 and mCherry-C1. All constructs were verified by sequencing (Base Clear, The Netherlands). HeLa cell line stably expressing H2A-GFP was described elsewhere [61], [62].

To express YFP-tagged mutant RAD18, cells were transfected with 2 µg of the pEYFP-mutantRAD18 plasmids using FuGene6 (Roche)

HeLa cells were cultured at 37°C in DMEM/F12 (GIBCO) supplemented with 5% v/v fetal calf serum, streptomycin sulfate and penicillin, under 5% CO_2_ in air. Female human primary fibroblast was cultured at 37°C in DMEM/F12 (GIBCO) supplemented with 15% v/v fetal calf serum, 1% non essential amino acid (GIBCO), 0.1 mM β-mercaptoethanol, and penicillin, under 5% CO_2_ in air.

### RAD18 mutants

All four putative ubiquitylation sites (K161, K261, K309, K318) were mutated to an alanine residue (Δubi). In the RING finger, C28 was mutated to phenylalanine (C28F), to abolish the E3 ligase activity [2]. In addition, a complete RING finger deletion mutant (ΔRING, aa25–63) was generated. Two different point mutations were generated in the Zinc finger; C207 was mutated to phenylalanine (C207F) [2], and a conserved aspartic acid D221, present in all Zinc finger domains that have been shown to bind to ubiquitin to date, was mutated to alanine (D221A). The whole Zinc finger (aa201–225) was also deleted (ΔZINC). In the SAP domain we generated combined mutations that have been shown to have a reduced binding activity for single-stranded and double-stranded DNA *in vitro* [14]. mSAP1 contained mutations at H263, G264, and L265, and mSAP2 was mutated at G269 and K271; all these residues were mutated to an alanine residue. The entire SAP domain (aa248–282) was also deleted (ΔSAP). In the HR6BD, A357 was mutated to arginine (A357R) to disrupt the binding to HR6A/B. Finally, a double mutation in the RING finger and the HR6A/B binding domain (C28F/A357R) was also generated.

### Antibodies

For primary antibodies, we used mouse monoclonal antibodies anti-GAPDH (Chemicon), anti-GFP (Roche), anti-RPA2 (Thermo Scientific), anti-FK2 (Millipore, #04-263), anti-PCNA PC10 (Abcam), anti-ubiquitylated histone H2A (Millipore, #05-678), and rabbit polyclonal antibodies anti-RAD18 [28], anti-GFP (Abcam), anti-phosphorylated CHK1 Ser-345 (Cell Signaling), anti-phosphorylated CHK2 at Tyr-68 (Cell Signaling), and anti-HR6A/B [78]. For secondary antibodies, we used a goat anti-rabbit/mouse IgG-peroxidase (Sigma), IgM-peroxidase (Sigma), IRDye680 goat-anti-rabbit/mouse IgG (Li-cor), IRDye800 goat-anti-rabbit/mouse IgG (Li-cor), goat anti-rabbit alexa 488/564, or goat anti-mouse alexa 488/564 (Molecular Probes). The antibody anti-GFP (Abcam) was also used to detect YFP, and was described in the text and figures as anti-YFP to avoid the confusion.

### RNA interference

Portions of 2×10^5^ cells were plated into six-well culture dishes. After 24 hours, cells were transfected with siRNA and incubated for 72 hours. Each transfection mixture contained 9 µl HiPerFect Transfection Reagent (Qiagen) and 2 µM siRNA (Ambion) in 400 µl serum-free DMEM/F12. siRNA28198 (si-endoRAD18) (Ambion) was used to downregulate only endogenous RAD18 but not YFP-RAD18 or mutant YFP-RAD18. siRPA1 (Ambion) [22] and siRPA2 (Ambion) were used to downregulate RPA70, and RPA34, respectively. The sequences of all siRNAs are shown in Table S4. A si-control (Ambion) which does not target any gene, was used as a control.

### Real-time RT-PCR

For real-time RT-PCR, RNA was prepared by Trizol from HeLa cells stably expressing shRAD18 or non-targeting shRNA, DNase-treated and reverse transcribed using random hexamer primers and Superscript II reverse transcriptase (Invitrogen). PCR was carried out with the iQ SYBR green PCR mastermix (Applied Biosystems) in the DNA engine Opticon 2 real-time PCR detection system (Bio-Rad) with RAD18 primer; forward/TCTGTATGCATGGGACAGGA, reverse/TCAGGTTCCAATTCCTCTGG. PCR of β-actin mRNA was included in each reaction and used to normalize the data. Three independent experiments were performed and each real-time PCR was performed in duplicate. All –RT reactions were negative.

### Global γ-ray irradiation

To induce global ionizing damage, cultured cells were irradiated with γ-rays from a _137_Cs source (0.71 Gy/minutes).

### Global UVC irradiation

To induce global UVC damage, cultured cells were irradiated with a Philips TUV lamp (254 nm) at a dose of 20 J/m^2^. For immunoblot analysis, cells were cultured for another 6 hours and whole-cell extracts were prepared.

### Protein immunoprecipitation analysis

HeLa cells expressing either wild type or mutant YFP-RAD18 were lysed in 2.0 ml Lysis buffer 1 (50 mM Tris–HCl pH 8.0, 450 mM NaCl, 5 mM MgCl_2_, 0.5 mM EDTA, 0.2% Nonidet P-40, 10% (v/v) glycerol, 0.5 mM dithiothreitol, and protease inhibitors (Roche)). The cell suspension was left on ice for 1 h, sonicated 5 times for 5 sec. Subsequently the lysate was treated with 0.125 U/µl of benzonase nuclease (Novagen) for 2 h, and centrifuged for 30 min (full speed at 4°C). The efficiency of nuclease was checked by DNA precipitation. The cell lysate was diluted with Lysis buffer 2 (50 mM Tris–HCl pH 8.0, 5 mM MgCl_2_, 0.5 mM EDTA, 0.2% Nonidet P-40, 10% (v/v) glycerol, 0.5 mM dithiothreitol, and protease inhibitors (Roche)) to decrease the concentration of NaCl to 150 mM. The cell lysate was pre-cleared with 20 µl of protein A-agarose beads (GE Healthcare) for 2 hours at 4°C. To cross-link antibodies with beads, antibodies (1 µg) were first incubated with 20 µl of beads for 2 hours at 4°C. The beads were washed three times with lysis buffer and subsequently incubated with borate buffer pH 8.0 containing 20 µM dimethyl pimelimidate (Sigma) for 1 hour at RT. The beads were washed once with ethanol amine pH 8.0 and incubated for 1 hour at RT. The beads were washed three times with lysis buffer and incubated with lysis buffer containing 5% BSA for 2 hours at 4°C. Subsequently, the beads were used for immunoprecipitation analysis. Each immunoprecipitation analysis contained 2 mg of lysate and 25 µl of protein A-agarose, and the mixture was incubated overnight at 4°C. After washing three times with 500 µl of Lysis buffer, bound proteins were analyzed by SDS–PAGE and immunoblots.

### Immunoblot analysis

Whole-cell extracts were prepared from cultured cells collected in a Tris buffer (50 mM Tris-HCl pH 6.8, 2% w/v SDS, 0.1% w/v bromophenol blue, 10% v/v glycerol and 100 mM dithiothreitol), and sonicated. After 5 minutes incubation at 100°C, the cell extracts were separated by SDS 10% polyacrylamide gel electrophoresis and transferred to nitrocellulose membranes. The membranes were incubated with antibodies to analyze expression of target proteins. The expression was detected using enhanced chemiluminescence (PerkinElmer). The target proteins detected using IRDye680 or IRDye800 were quantified with Odyssey (Li-cor).

### Immunocytochemistry

Cells were cultured on 24 mm round coverslips, washed with PBS and fixed in 2% w/v paraformaldehyde in PBS for 15 minutes. To detect ubiquitylation of histone H2A, cells were pretreated with 0.2% v/v Triton X-100 in PBS for 30 seconds before the fixation step. Cells were permeabilized for 20 minutes with 0.2% v/v Triton X-100 in PBS. Cells were blocked with PBS^+^ (PBS containing 0.5% w/v BSA and 0.15% w/v glycine) for 30 minutes, incubated with primary antibody for 2 hours, and washed with 0.2% Triton X-100 in PBS. Subsequently, cells were incubated with secondary antibody for 1 hour, washed with 0.2% Triton X-100 in PBS. Coverslips were placed on a slide, mounted with Prolong Gold reagent (Invitrogen).

### Confocal and time-lapse microscopy

Images of living cells expressing YFP-tagged and mCherry-tagged proteins were obtained using a Zeiss LSM510NLO microscope (Carl Zeiss) with a 63×/1.40 NA oil immersion lens. Cells were maintained at 37°C in a mixture of air with 5% CO_2_. YFP-tagged proteins were detected by exciting YFP with a 488 nm Argon gas laser and monitoring YFP emission through a 500–550 band-pass filter. mCherry-tagged proteins were detected by exciting mCherry with a 543 nm helium neon laser and monitoring mCherry emission through a long-pass 560 filter. To minimize the effect of photo-bleaching, images were taken with 10 µW for a 488 nm laser, and with 20 µW for a 543 nm laser. For time-lapse experiments, cell images of 6 confocal planes at 1.5 µm intervals were taken every 20 minutes for 27 hours. All images were captured with a line average of 2. Time-lapse images were analysed using the ImageJ software (Rasband, W.S., ImageJ, U.S. National Institutes of Health, Bethesda, Maryland, USA [http://rsb.info.nih.gov/ij/]).

### Local multi-photon DNA damage induction

A Coherent Mira mode-locked Ti:Sapphire laser (multi-photon laser, MPL) (Coherent) connected to a Zeiss LSM510NLO confocal microscope was used at 800 nm with a pulse length of 200 fs and repetition rate of 76 MHz. For local DNA damage induction, an area of irradiation was set at 4 µm^2^ (40×40 pixels), and the output of laser power was set at 75 mW at pixel-dwell time 1.6 µs with 5 iterations. Two images were taken before MPL irradiation and monitored at 10 seconds intervals for 10 minutes immediately after MPL irradiation. All images were captured with a line average of 1.

### Yeast two-hybrid analysis

*Saccharomyces cerevisiae* AH109 (Clonetech) were cultured in YPD medium (1% (w/v) yeast extract (Difco), 2% peptone (w/v) 2% glucose (w/v)) at 30°C. When the density of the medium was 1.0, cells were harvested and washed twice with distilled water, mixed with transformation buffer (240 µl 50% v/v PEG1500, 36 µl 1 M LiAc), 5 µl 10 mM salmon sperm DNA (Clontech), 50 µl H_2_O, 4 µg of pGADT7 vector carrying target DNA with Gal4 activation domain, and 4 µg of pGBKT7 carrying target DNA with Gal4 binding domain), incubated 30 min at 30°C, and subsequently 30 min at 42°C. The transformation buffer was discarded and 10% of transformed cells were spread onto the selection drop medium SD-L-W (Clontech). Interactions were determined by spot assay on SD-L-W-H (Clontech) and SD-L-W-H-A (Clontech) indicator plates.

## Supporting Information

Figure S1 **Knockdown of** ***RAD18*** **and functional analyses of** ***RAD18*** **mutants.** (A) Transient downregulation of RAD18 with siRNA against Rad18 in Hela cells (si-RAD18) stably expressing GFP-RAD9 and transiently co-expressing mCherry-PCNA. (B) The level of RAD18 mRNA and protein expression in three different HeLa cell lines stably expressing shRNA targeting *RAD18*. The control level, in HeLa cells stably expressing non-targeting shRNA, was set at 1.0. (C) Expression of endogenous RAD18 was detected by immunostaining using anti-RAD18 in control and RAD18 knockdown cell lines (shRAD18). Cells were irradiated with IR (5 Gy), and fixed after 1 hour. (D) Analyses of RAD18 ubiquitylation. Endogenous RAD18 was transiently knocked down in HeLa cells stably expressing YFP-RAD18 or transiently expressing YFP-RAD18Δubi. Cells were lysed (input) and immunoprecipitation with YFP antibody (IP αYFP) was performed. The expression levels of RAD18 and HR6A/B in the lysate are shown as input. Immunoprecipitated RAD18 and co-immunoprecipitated HR6A/B were detected on immunoblots (IP αYFP). (E) Endogenous RAD18 was transiently knocked down (si-endoRAD18) in HeLa cells transiently expressing YFP-RAD18 mutated in putative auto-ubiquitylation sites. Auto-ubiquitylation of YFP-RAD18 was analyzed on immunoblots. GAPDH was used as a loading control. (F) Endogenous RAD18 was transiently knocked down (si-endoRAD18) in HeLa cells stably expressing YFP-RAD18 or transiently expressing YFP-mutant RAD18. Cells were irradiated with 20 J/m^2^ UVC, and whole-cell extracts were prepared 8 h after irradiation. Expression levels of mono-ubiquitylated PCNA were analyzed on immunoblots using αPCNA. Arrowheads point at mono-ubiquitylated PCNA (ubi-PCNA). The panels show the results of a representative experiment, where three independent experiments yielded similar results. (G) Cell lysates from wild type HeLa cells and HeLa cells stably expressing YFP-RAD18 were treated with or without benzonase nuclease for 2 h on ice. In the presence of benzonase, DNA was absent from the cell lysates. M in the figure indicates DNA ladder.
(TIF)

Figure S2 **Subnuclear localization of wild type and mutant YFP-RAD18 during the cell cycle and after irradiation.** Confocal images of living HeLa cells expressing wild-type or mutant YFP-RAD18 in G1, S, and late G2 phases, and after irradiation with IR (5 Gy). Endogenous RAD18 was downregulated by siRNA (si-endoRAD18). RAD18 carrying mutations in its Zinc finger (C207F, D221A) and a deletion of the Zinc finger (ΔZINC) showed no cell cycle specific localization. In contrast, RAD18 carrying either mutations of the RING finger (C28F, ΔRING) or SAP domain (mSAP1, mSAP2, ΔSAP) showed a localization pattern identical to that of wild-type RAD18 (WT).
(TIF)

Figure S3 **No direct interaction between RAD18 and components of the 9-1-1 complex.** Yeast two-hybrid assay. Yeast carrying both pGADT7-target DNA and pGBKT7-target DNA were spotted on SD-L-W plates to confirm the yeast transformation. The interaction was confirmed by growth on selective medium plates, SD-L-W+X-Gal, SD-L-W-H and SD-L-W-H-A. The protein expression of transformed plasmids was examined on immunoblots by different antibodies as indicated. pGADT7 vector contains a HA-epitope tag, and the pGBKT7 vector contains a c-myc epitope tag. Three independent experiments were performed and representative results are shown. A) Yeast two hybrid assay between RAD18 and all 9-1-1 components. Various types of DNA damage were induced by irradiation with 50 Gy, or growth on the selective medium plates containing 25 mM HU, 0.05% MMS, or 8 µM CPT. Different concentrations of HU (6.25 mM, 12.5 mM, 50 mM, 100 mM), MMS (0.005%, 0.01%, 0.1%, 0.2%), CPT (2 µM, 4 µM, 16 µM, 32 µM) were examined and showed similar results (data not shown). B) Yeast two hybrid assay between the 9-1-1 components. C) Yeast two hybrid assay between RAD18, and HR6A, HR6B or PCNA.
(TIF)

Figure S4 **Normal CHK1 and CHK2 phosphorylation in** ***RAD18*** **knockdown Hela cells.** HeLa cells stably expressing non-targeting shRNA or shRNA targeting RAD18 were exposed either with UV at 20 J/m^2^ or IR at 10 Gy. Prior to irradiation, and after certain time points indicated in the figure, phosphorylation of CHK1 at Ser 354 and CHK2 at Tyr 68 was analyzed on immunoblots. GAPDH was used as a loading control.
(TIF)

Movie S1 **Analyses of wild type RAD18 localization during the cell cycle.** Time lapse images of nuclei expressing wild type YFP-RAD18. Images were captured every 30 min for 27 hours, a single nucleus was selected, aligned, and 5 frames of the images are shown per second. Endogenous RAD18 was downregulated by siRNA. Analyses began in S phase, and the cell went through G2, M and G1. The growth rates of wild type and mutant cells were not different (data not shown).
(AVI)

Movie S2 **Analyses of mutant RAD18 localization during G1, S and G2 phase.** Time lapse images of nuclei expressing YFP-RAD18 carrying a mutation in its Zinc finger (D221A). Images were captured every 30 min for 27 hours, a single nucleus was selected, aligned, and 5 frames of the images are shown per second. Endogenous RAD18 was downregulated by siRNA. Analyses started in G1 phase, and the cell went through S, and most likely G2. The cell cycle phases of mutant YFP-D221A were inferred from the time passed since the previous M-phase or the time span until the next M-phase, and the duration of G1, S, G2 and M phase in wild type cells. The growth rates of wild type and mutant cells were not different (data not shown).
(AVI)

Movie S3 **Analyses of mutant RAD18 localization during M, G1 and S phase.** Time lapse images of nuclei expressing YFP-RAD18 carrying a mutation in its Zinc finger (D221A). Images were captured every 30 min for 27 hours, a single nucleus was selected, aligned, and 5 frames of the images are shown per second. Endogenous RAD18 was downregulated by siRNA. Analyses began in G2 phase and the cell went through, M, G1, and most likely S. The cell cycle phases of mutant YFP-D221A were inferred from the time passed since the previous M-phase or the time span until the next M-phase, and the duration of G1, S, G2 and M phase in wild type cells. The growth rates of wild type and mutant cells were not different (data not shown).
(AVI)

Table S1 **Designed primers to create mutant RAD18.** Mutated amino acid codons are shown in red, and the introduced mutations are shown in bold italics
(DOC)

Table S2 **Primers used to generate Y2H vectors.** Restriction enzyme sites used for subcloning are underlined.
(DOC)

Table S3 **Primers used to generate a vector carrying the shRAD18 sequence.**
(DOC)

Table S4 **siRNAs used in this study.**
(DOC)
