# Supplementary figures and images for: Human RAD18 Interacts with Ubiquitylated Chromatin Components and Facilitates RAD9 Recruitment to DNA Double Strand Breaks
Source: PLoS One. 2011 Aug 17;6(8):e23155. doi: 10.1371/journal.pone.0023155 (PMC3157352; doi:10.1371/journal.pone.0023155)

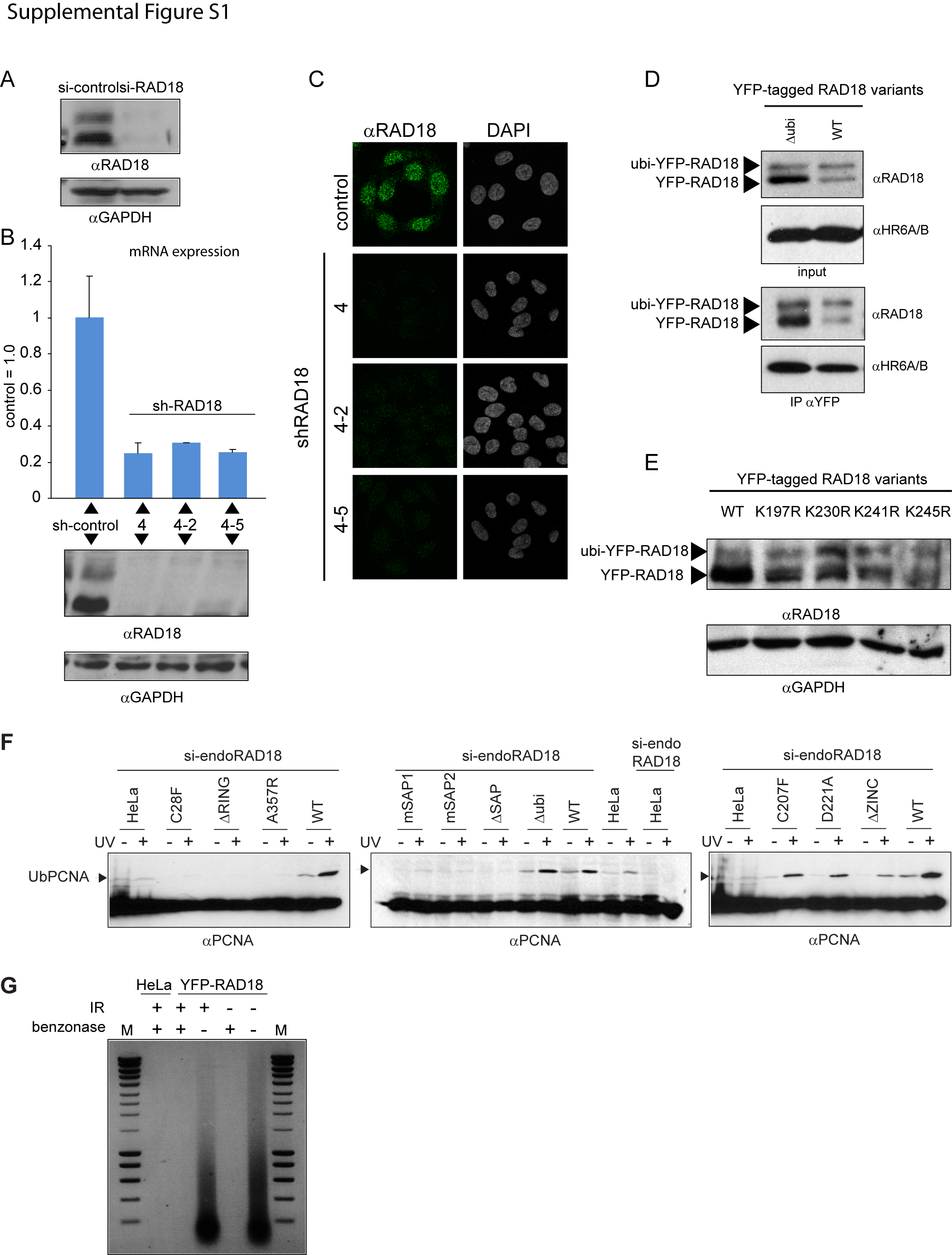

Supplement: Figure S1 — Knockdown of RAD18 and functional analyses of RAD18 mutants. (A) Transient downregulation of RAD18 with siRNA against Rad18 in Hela cells (si-RAD18) stably expressing GFP-RAD9 and transiently co-expressing mCherry-PCNA. (B) The level of RAD18 mRNA and protein expression in three different HeLa cell lines stably expressing shRNA targeting RAD18. The control level, in HeLa cells stably expressing non-targeting shRNA, was set at 1.0. (C) Expression of endogenous RAD18 was detected by immunostaining using anti-RAD18 in control and RAD18 knockdown cell lines (shRAD18). Cells were irradiated with IR (5 Gy), and fixed after 1 hour. (D) Analyses of RAD18 ubiquitylation. Endogenous RAD18 was transiently knocked down in HeLa cells stably expressing YFP-RAD18 or transiently expressing YFP-RAD18Δubi. Cells were lysed (input) and immunoprecipitation with YFP antibody (IP αYFP) was performed. The expression levels of RAD18 and HR6A/B in the lysate are shown as input. Immunoprecipitated RAD18 and co-immunoprecipitated HR6A/B were detected on immunoblots (IP αYFP). (E) Endogenous RAD18 was transiently knocked down (si-endoRAD18) in HeLa cells transiently expressing YFP-RAD18 mutated in putative auto-ubiquitylation sites. Auto-ubiquitylation of YFP-RAD18 was analyzed on immunoblots. GAPDH was used as a loading control. (F) Endogenous RAD18 was transiently knocked down (si-endoRAD18) in HeLa cells stably expressing YFP-RAD18 or transiently expressing YFP-mutant RAD18. Cells were irradiated with 20 J/m2 UVC, and whole-cell extracts were prepared 8 h after irradiation. Expression levels of mono-ubiquitylated PCNA were analyzed on immunoblots using αPCNA. Arrowheads point at mono-ubiquitylated PCNA (ubi-PCNA). The panels show the results of a representative experiment, where three independent experiments yielded similar results. (G) Cell lysates from wild type HeLa cells and HeLa cells stably expressing YFP-RAD18 were treated with or without benzonase nuclease for 2 h on ice. In the pre [file pone.0023155.s001.tif]

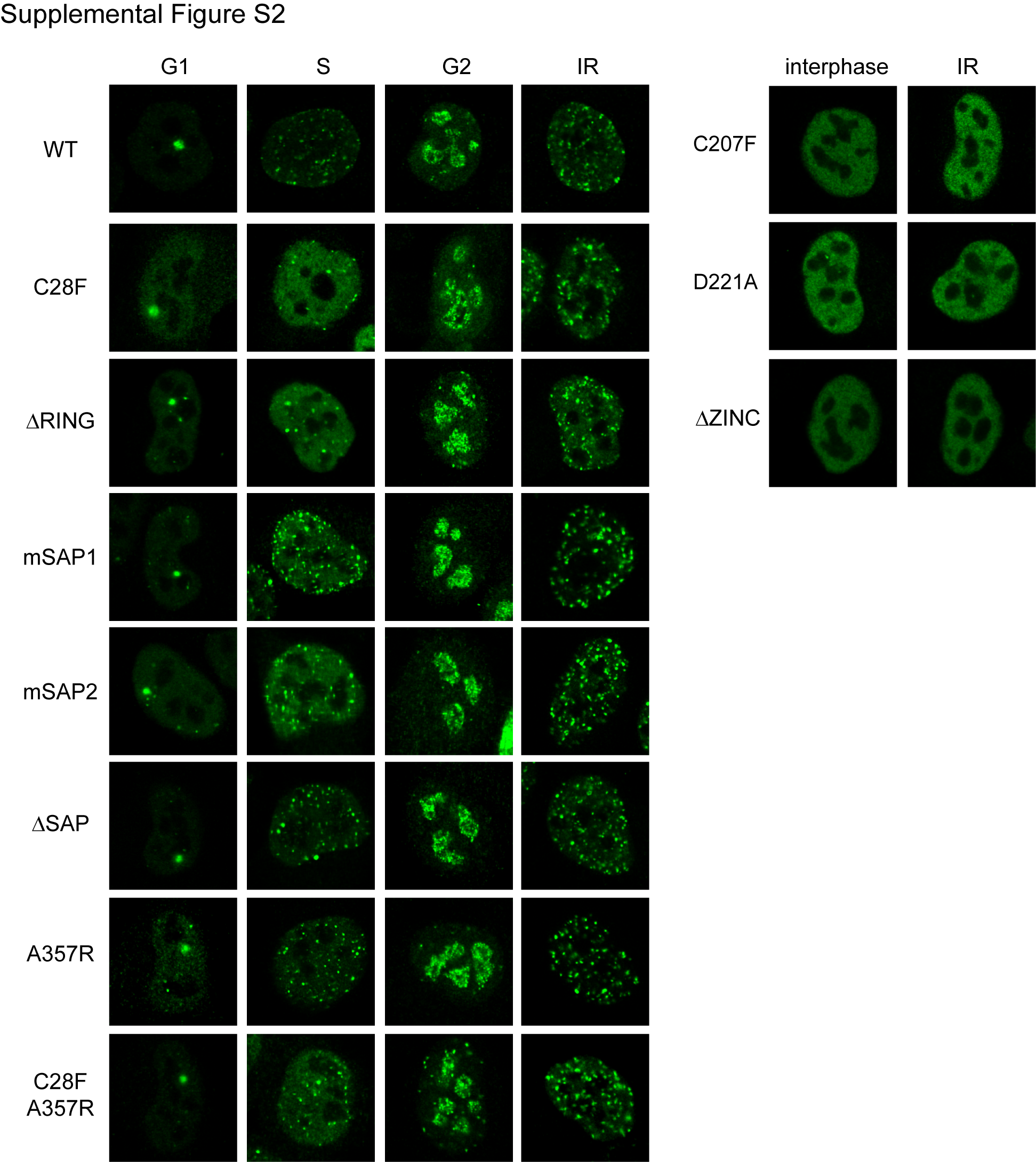

Supplement: Figure S2 — Subnuclear localization of wild type and mutant YFP-RAD18 during the cell cycle and after irradiation. Confocal images of living HeLa cells expressing wild-type or mutant YFP-RAD18 in G1, S, and late G2 phases, and after irradiation with IR (5 Gy). Endogenous RAD18 was downregulated by siRNA (si-endoRAD18). RAD18 carrying mutations in its Zinc finger (C207F, D221A) and a deletion of the Zinc finger (ΔZINC) showed no cell cycle specific localization. In contrast, RAD18 carrying either mutations of the RING finger (C28F, ΔRING) or SAP domain (mSAP1, mSAP2, ΔSAP) showed a localization pattern identical to that of wild-type RAD18 (WT). (TIF) [file pone.0023155.s002.tif]

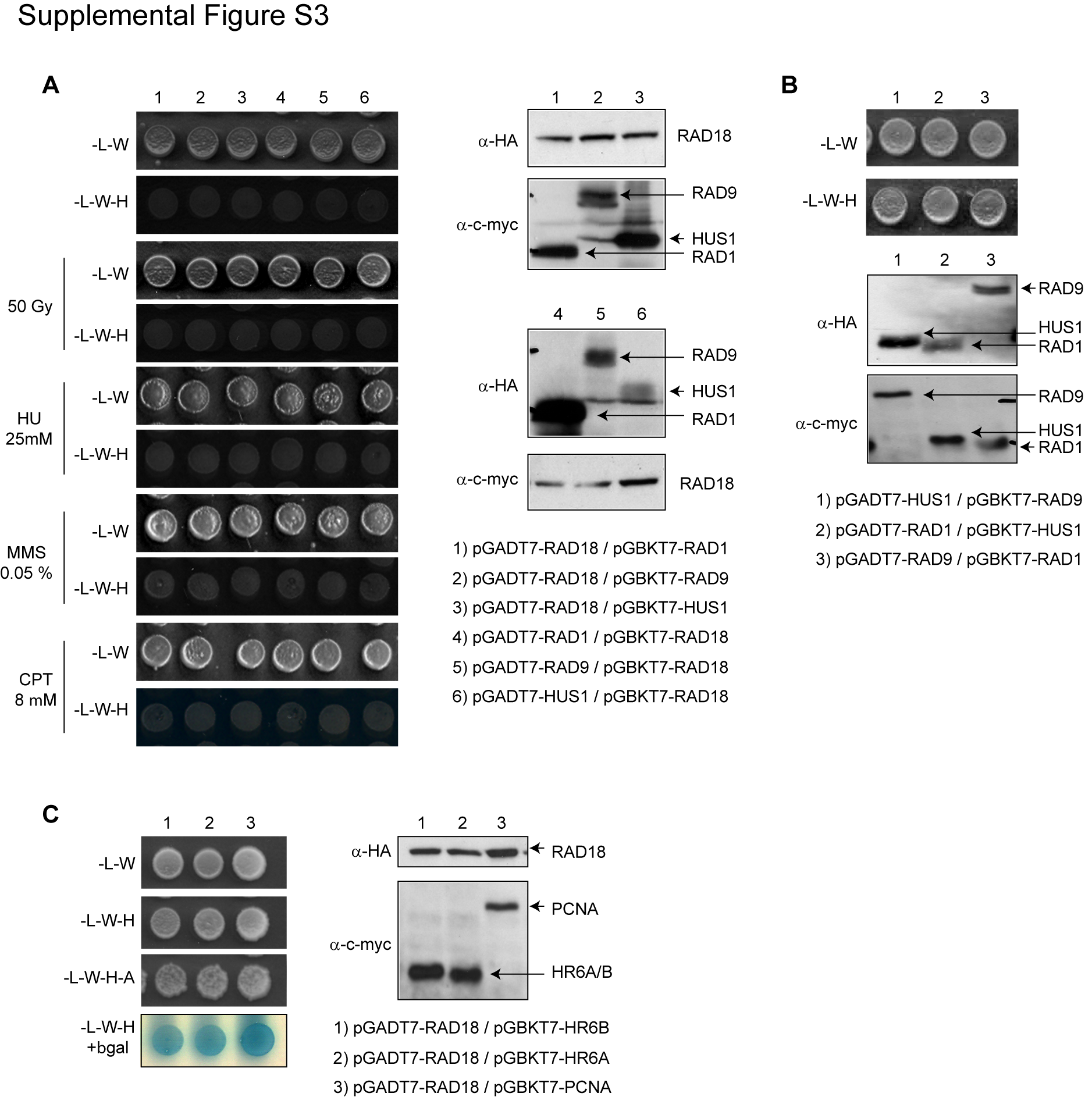

Supplement: Figure S3 — No direct interaction between RAD18 and components of the 9-1-1 complex. Yeast two-hybrid assay. Yeast carrying both pGADT7-target DNA and pGBKT7-target DNA were spotted on SD-L-W plates to confirm the yeast transformation. The interaction was confirmed by growth on selective medium plates, SD-L-W+X-Gal, SD-L-W-H and SD-L-W-H-A. The protein expression of transformed plasmids was examined on immunoblots by different antibodies as indicated. pGADT7 vector contains a HA-epitope tag, and the pGBKT7 vector contains a c-myc epitope tag. Three independent experiments were performed and representative results are shown. A) Yeast two hybrid assay between RAD18 and all 9-1-1 components. Various types of DNA damage were induced by irradiation with 50 Gy, or growth on the selective medium plates containing 25 mM HU, 0.05% MMS, or 8 µM CPT. Different concentrations of HU (6.25 mM, 12.5 mM, 50 mM, 100 mM), MMS (0.005%, 0.01%, 0.1%, 0.2%), CPT (2 µM, 4 µM, 16 µM, 32 µM) were examined and showed similar results (data not shown). B) Yeast two hybrid assay between the 9-1-1 components. C) Yeast two hybrid assay between RAD18, and HR6A, HR6B or PCNA. (TIF) [file pone.0023155.s003.tif]

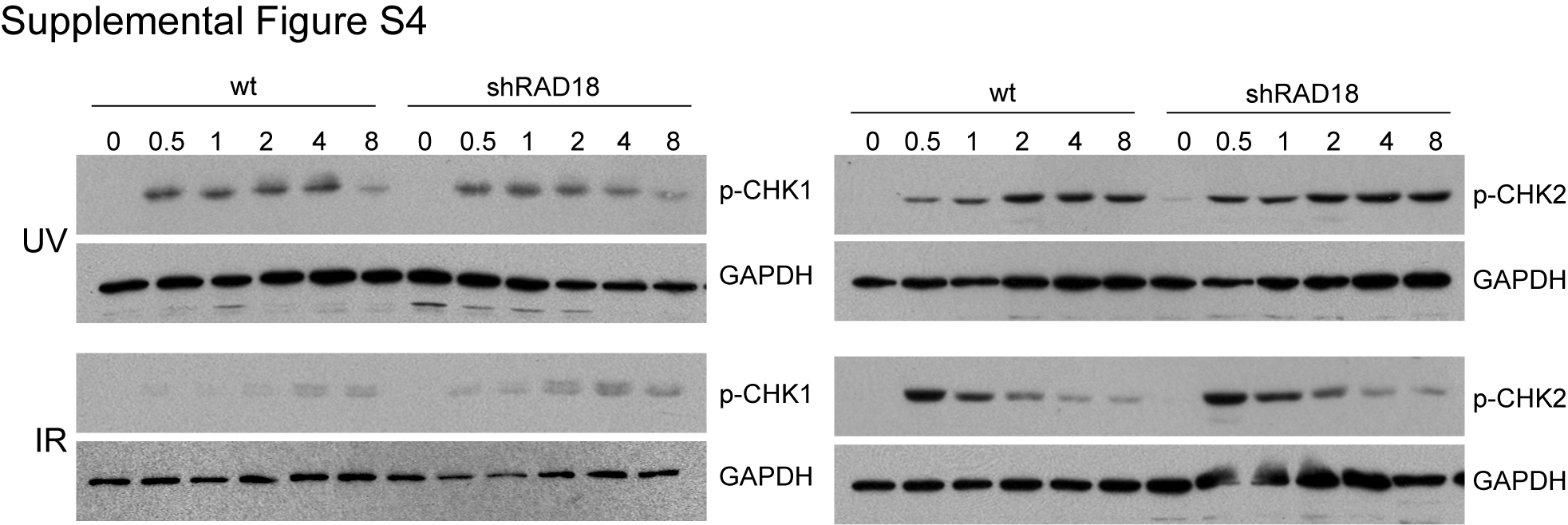

Supplement: Figure S4 — Normal CHK1 and CHK2 phosphorylation in RAD18 knockdown Hela cells. HeLa cells stably expressing non-targeting shRNA or shRNA targeting RAD18 were exposed either with UV at 20 J/m2 or IR at 10 Gy. Prior to irradiation, and after certain time points indicated in the figure, phosphorylation of CHK1 at Ser 354 and CHK2 at Tyr 68 was analyzed on immunoblots. GAPDH was used as a loading control. (TIF) [file pone.0023155.s004.tif]
