# Supplementary material for: Human RAD18 Interacts with Ubiquitylated Chromatin Components and Facilitates RAD9 Recruitment to DNA Double Strand Breaks
Source: PLoS One. 2011 Aug 17;6(8):e23155. doi: 10.1371/journal.pone.0023155 (PMC3157352; doi:10.1371/journal.pone.0023155)
Supplement: Table S1 — Designed primers to create mutant RAD18. Mutated amino acid codons are shown in red, and the introduced mutations are shown in bold italics (DOC) [file pone.0023155.s008.doc]

**Supplementary Table S1**

Designed primers to create mutant RAD18

| RING finger aa 25-63 | |
| --- | --- |
| C28F | |
| forward | GATTTGCTGCGGTGTGGAATTT***T***CTTCGAGTATTTCAACATTGC* |
| reverse | GCAATGTTGAAATACTCGAAGAAAATTCCACACCGCAGCAAATC |
| Deletion | |
| forward | CAATAGATGATTTGCTGCGGTGTGTGACTGTCACAGAG |
| reverse | CTCTGTGACAGTCACACACCGCAGCAAATCATCTATTG |
| Zinc finger aa 201-225 | |
| C207F | |
| forward | CTAAAGTGGATTGTCCTGTTT***T***CGGGGTTAACATTCCAGAAAG |
| reverse | CTTTCTGGAATGTTAACCCCGAAAACAGGACAATCCACTTTAG |
| D221A | |
| forward | GTCACATTAATAAGCATTTAG***C***CAGCTGTTTATCACGCGAAGA |
| reverse | TCTTCGCGTGATAAACAGCTGGCTAAATGCTTATTAATGTGAC |
| Deletion | |
| forward | CCACTTTGAAACAAGTTACTCGCGAAGAGAAGAAGGAAAG |
| reverse | CTTTCCTTCTTCTCTTCTCGAGTAACTTGTTTCAAAGTGG |
| SAP domain aa 248-282 | |
| G269A+K271A (mSAP1) | |
| forward | GCATGGATTATCTATTCAAG***C***AAAT***GC***ACAACAGCTCATTAAAAGGC |
| reverse | GCCTTTTAATGAGCTGTTGTGCATTTGCTTGAATAGATAATCCATGC |
| H263A+G264A+L265 (mSAP2) | |
| forward | GAAAAAGCTAAAAGAG***GC***TG***C***A***GC***ATCTATTCAAGGAAATAAACAACAGC |
| reverse | GCTGTTGTTTATTTCCTTGAATAGATGCTGCAGCCTCTTTTAGCTTTTTC |
| Deletion | |
| forward | GCTGCCCAAAACTGTACACATGTACAATGCCCAATG |
| reverse | CATTGGGCATTGTACATGTGTACAGTTTTGGGCAGC |
| Auto-ubiquitylation sites | |
| K161R | |
| forward | CAAATTCAGCCCTCAAA***G***AGAGGCGAGCCCTGCTG |
| reverse | CAGCAGGGCTCGCCTCTCTTTGAGGGCTGAATTTG |
| K197R | |
| forward | CACCCTCGACATCCACTTTGA***G***ACAAGTTACTAAAGTGGATTG |
| reverse | CAATCCACTTTAGTAACTTGTCTCAAAGTGGATGTCGAGGGTG |
| K230R | |
| forward | GTTTATCACGCGAAGAGAAGA***G***GGAAAGCCTCAGAAGTTCTG |
| reverse | CAGAACTTCTGAGGCTTTCCCTCTTCTCTTCGCGTGATAAAC |
| K241R | |
| forward | GAAGTTCTGTTCACAAAAGGA***G***GCCGCTGCCCAAAACTG |
| reverse | CAGTTTTGGGCAGCGGCCTCCTTTTGTGAACAGAACTTC |
| K245R | |
| forward | CAAAAGGAAGCCGCTGCCCA***G***AACTGTATATAATTTGCTCTC |
| reverse | GAGAGCAAATTATATACAGTTCTGGGCAGCGGCTTCCTTTTG |
| K261R | |
| forward | CGTGATTTAAAGAAAAAGCTAA***G***AGAGCATGGATTATCTATTCAAG |
| reverse | CTTGAATAGATAATCCATGCTCTCTTAGCTTTTTCTTTAAATCACG |
| K309R | |
| forward | GAAATCGAAAATATAGAGA***G***GACTAGGATGCGTCTTGAAGC |
| reverse | GCTTCAAGACGCATCCTAGTCCTCTCTATATTTTCGATTTC |
| K318R | |
| forward | GGATGCGTCTTGAAGCTAGTA***G***ACTCAATGAAAGTGTAATGG |
| reverse | CCATTACACTTTCATTGAGTCTACTAGCTTCAAGACGCATCC |
| HR6A/B binding domain | |
| A357R | |
| forward | GAATTTCAGCTTCTGGTGGATCAG***CG***TAGAAAAGGATACAAGAAAATTG |
| reverse | CAATTTTCTTGTATCCTTTTCTACGCTGATCCACCAGAAGCTGAAATTC |
| Silent mutation against shRAD18 | |
| forward | CACGCGAAGAGAAGAAGGA***G***AG***T***CT***G***AG***G***AG***C***TCTGTTCACAAAAGGAAGCCG |
| reverse | CGGCTTCCTTTTGTGAACAGAGCTCCTCAGACTCTCCTTCTTCTCTTCGCGTG |

* Mutated amino acid codons are shown in red, and the introduced mutations are shown in bold italics.
