# Supplementary material for: Human RAD18 Interacts with Ubiquitylated Chromatin Components and Facilitates RAD9 Recruitment to DNA Double Strand Breaks
Source: PLoS One. 2011 Aug 17;6(8):e23155. doi: 10.1371/journal.pone.0023155 (PMC3157352; doi:10.1371/journal.pone.0023155)
Supplement: Table S2 — Primers used to generate Y2H vectors. Restriction enzyme sites used for subcloning are underlined. (DOC) [file pone.0023155.s009.doc]

**Supplementary Table S2**

Primers used to generate Y2H vectors

| *RAD18* | |
| --- | --- |
| forward | GAATTCGACTCCCTGGCCGAGTCTC (*Eco*RI)* |
| reverse | GGATCCTTAATTCCTATTACGCTTGTTTCT (*Bam*HI) |
| *PCNA* | |
| forward | CATATGTTCGAGGCGCGCCTGG (*Nde*I) |
| reverse | GGATCCCTAAGATCCTTCTTCATCCTCG (*Bam*HI) |
| *RAD9* | |
| forward | CATATGAAGTGCCTGGTCACGGGCG (*Nde*I) |
| reverse | GGATCCTCAGCCTTCACCCTCACTGT (*Bam*HI) |
| *HUS1* | |
| forward | CATATGAAGTTTCGGGCCAAGATCGTG (*Nde*I) |
| reverse | GGATCCCTAGGACAGCGCAGGGATGA (*Bam*HI) |
| *RAD1* | |
| forward | CATATGCCCCTTCTGACCCAACAGAT (*Nde*I) |
| reverse | GGATCCTCAAGACTCAGATTCAGGAACTT (*Bam*HI) |
| *Hr6a* | |
| forward | CATATGTCGACCCCGGCCCGG (*Nde*I) |
| reverse | GGATCCTCAACAGTCGCGCCAGCTTT (*Bam*HI) |
| *Hr6b* | |
| forward | GAATTCATGTCGACCCCGGCCCGT (*Eco*RI) |
| reverse | GGATCCTTATGAATCATTCCAGCTTTGC (*Bam*HI) |

* Restriction enzyme sites used for subcloning are underlined
