# Supplementary material for: Human RAD18 Interacts with Ubiquitylated Chromatin Components and Facilitates RAD9 Recruitment to DNA Double Strand Breaks
Source: PLoS One. 2011 Aug 17;6(8):e23155. doi: 10.1371/journal.pone.0023155 (PMC3157352; doi:10.1371/journal.pone.0023155)
Supplement: Table S3 — Primers used to generate a vector carrying the shRAD18 sequence. (DOC) [file pone.0023155.s010.doc]

**Supplementary Table S3**

Primers used to generate a vector carrying the shRAD18 sequence

| shRAD18 | |
| --- | --- |
| forward | GATCCCCGGAAAGCCTCAGAAGTTCTTTCAAGAGAAGAACTTCTGAGGCTTTCCTTTTTC |
| reverse | TCGAGAAAAAGGAAAGCCTCAGAAGTTCTTCTCTTGAAAGAACTTCTGAGGCTTTCCGGG |
