# Supplementary material for: Human RAD18 Interacts with Ubiquitylated Chromatin Components and Facilitates RAD9 Recruitment to DNA Double Strand Breaks
Source: PLoS One. 2011 Aug 17;6(8):e23155. doi: 10.1371/journal.pone.0023155 (PMC3157352; doi:10.1371/journal.pone.0023155)
Supplement: Table S4 — siRNAs used in this study. (DOC) [file pone.0023155.s011.doc]

**Supplementary Table S4**

siRNAs used in this study

| endoRAD18 | GGAAAUAGAUGAAAUCCAC |
| --- | --- |
| RPA1 | CACUCUAUCCUCUUUCAUG |
| RPA2 | CCUAGUUUCACAAUCUGU |
